# Supplementary material for: Probabilistic mathematical modelling to predict the red cell phenotyped donor panel size
Source: PLoS One. 2022 Nov 11;17(11):e0276780. doi: 10.1371/journal.pone.0276780 (PMC9651592; doi:10.1371/journal.pone.0276780)
Supplement: S1 Table — (DOCX) [file pone.0276780.s001.docx]

## Antigen Combination Analysis

S 1 Table. Antigen combinations that are met only by extended phenotyping.

| 1. Antigen combination data specific to extended phenotyping | | | | | | | |
| --- | --- | --- | --- | --- | --- | --- | --- |
| Antigen combination | Total num. req. units | Total num. of req. | Fraction  of  27001 | Antigen combination | Total num. req. units | Total num. of req. | Fraction of 27001 |
| c-E-K- | 30673 | 11280 | 0.41776 | C-E-Fya-K-s- | 11 | 6 | 0.00022 |
| C-E-K- | 10346 | 4409 | 0.16329 | C-E-Fyb-K-S- | 13 | 6 | 0.00022 |
| E-K- | 3711 | 1235 | 0.04574 | c-Fya-K- | 23 | 6 | 0.00022 |
| C-e-K- | 1459 | 590 | 0.02185 | C-Fyb-Jkb-K- | 8 | 6 | 0.00022 |
| K-M- | 707 | 352 | 0.01304 | C-Jkb-K-S- | 13 | 6 | 0.00022 |
| E-Fya-K- | 1004 | 338 | 0.01252 | E-Fyb-Jkb-K-s- | 6 | 6 | 0.00022 |
| Fya-K- | 810 | 334 | 0.01237 | E-M- | 12 | 6 | 0.00022 |
| C-E-Fya-Jkb-K- | 604 | 318 | 0.01178 | Fya-K-M-S- | 10 | 6 | 0.00022 |
| K-S- | 1041 | 308 | 0.01141 | Fyb-Jkb-K- | 11 | 6 | 0.00022 |
| Jka-K- | 673 | 301 | 0.01115 | Jka-K-S- | 13 | 6 | 0.00022 |
| c-E-Jka-K- | 748 | 297 | 0.011 | Jkb-S- | 15 | 6 | 0.00022 |
| C-K- | 659 | 294 | 0.01089 | M-S- | 14 | 6 | 0.00022 |
| c-E-Fya-K- | 689 | 282 | 0.01044 | C-e-k- | 8 | 5 | 0.00019 |
| E-Jka-K- | 653 | 270 | 0.01 | c-E-M- | 10 | 5 | 0.00019 |
| Jka- | 461 | 206 | 0.00763 | C-Fya-Jkb-K- | 5 | 5 | 0.00019 |
| K- | 554 | 204 | 0.00756 | c-Jka-K- | 13 | 5 | 0.00019 |
| Fya- | 499 | 196 | 0.00726 | C-K-s- | 11 | 5 | 0.00019 |
| c-E-Jkb-K- | 383 | 184 | 0.00681 | E-Fyb- | 16 | 5 | 0.00019 |
| C-E-Fya-K- | 430 | 183 | 0.00678 | E-Fyb-Jkb-K-M-S- | 6 | 5 | 0.00019 |
| c-E- | 435 | 160 | 0.00593 | C-E-Fya-K-M-S- | 8 | 4 | 0.00015 |
| E-Jkb-K- | 419 | 159 | 0.00589 | C-E-Fyb-Jka-K-S- | 9 | 4 | 0.00015 |
| M- | 367 | 156 | 0.00578 | C-Fya-Jka-K-S- | 7 | 4 | 0.00015 |
| C-E-Jka-K- | 329 | 142 | 0.00526 | C-Fya-Jkb-S- | 9 | 4 | 0.00015 |
| C-Fya-K- | 319 | 142 | 0.00526 | C-S- | 13 | 4 | 0.00015 |
| c-E-Fyb-Jka-K- | 299 | 132 | 0.00489 | E-Fya-Jka-S- | 5 | 4 | 0.00015 |
| C-e- | 340 | 119 | 0.00441 | E-Fya-Jkb- | 9 | 4 | 0.00015 |
| Jkb-K- | 274 | 110 | 0.00407 | e-Jka-K- | 10 | 4 | 0.00015 |
| c-E-Fyb-Jkb-K- | 219 | 108 | 0.004 | E-Jka-K-M-S- | 10 | 4 | 0.00015 |
| c-E-Fyb-K- | 214 | 104 | 0.00385 | E-Jkb-K-M- | 6 | 4 | 0.00015 |
| C-E-Jkb-K- | 215 | 96 | 0.00356 | Fya-Jka-K-M-S- | 6 | 4 | 0.00015 |
| E-Fya-Jkb-K- | 276 | 96 | 0.00356 | Fya-Jkb-K-S- | 8 | 4 | 0.00015 |
| E-Fyb-Jkb-K- | 203 | 92 | 0.00341 | Fya-M-S- | 7 | 4 | 0.00015 |
| E- | 222 | 87 | 0.00322 | Jka-M- | 4 | 4 | 0.00015 |
| E-K-S- | 215 | 80 | 0.00296 | C-E-Fya-Jka-K-S- | 4 | 3 | 0.00011 |
| c-E-Fya-Jkb-K- | 200 | 79 | 0.00293 | C-E-Fya-K-M- | 7 | 3 | 0.00011 |
| C-Jka-K- | 187 | 79 | 0.00293 | c-E-Fyb- | 7 | 3 | 0.00011 |
| c-K- | 191 | 77 | 0.00285 | c-E-Fyb-K-M-S- | 4 | 3 | 0.00011 |
| E-Fyb-K- | 196 | 76 | 0.00281 | c-E-Jka-K-M- | 4 | 3 | 0.00011 |
| C-Jkb-K- | 191 | 74 | 0.00274 | C-e-Jka-K-S- | 6 | 3 | 0.00011 |
| E-Fya-Jka-K- | 201 | 69 | 0.00256 | C-e-Jkb-K-S- | 5 | 3 | 0.00011 |
| C-e-Jka-K- | 143 | 68 | 0.00252 | C-E-S- | 7 | 3 | 0.00011 |
| E-K-M- | 159 | 68 | 0.00252 | C-Fya-Jkb- | 5 | 3 | 0.00011 |
| Fya-Jkb-K- | 160 | 63 | 0.00233 | c-Fyb-K- | 6 | 3 | 0.00011 |
| E-Jka-K-S- | 130 | 62 | 0.0023 | c-Fyb-K-S- | 9 | 3 | 0.00011 |
| C-E- | 149 | 60 | 0.00222 | C-Jka-K-M-S- | 6 | 3 | 0.00011 |
| C-E-Fya-K-S- | 159 | 60 | 0.00222 | C-Jka-K-S- | 6 | 3 | 0.00011 |
| Fyb-Jka-K- | 114 | 60 | 0.00222 | c-K-M- | 4 | 3 | 0.00011 |
| C-E-K-S- | 116 | 59 | 0.00219 | E-Fya-Jka-K-M-S- | 6 | 3 | 0.00011 |
| C-E-Fya-Jkb-K-S- | 155 | 58 | 0.00215 | E-Fya-Jka-K-s- | 5 | 3 | 0.00011 |
| c-E-Fya-K-S- | 119 | 55 | 0.00204 | E-Fya-k- | 4 | 3 | 0.00011 |
| E-Fya-K-S- | 126 | 55 | 0.00204 | E-Fya-s- | 6 | 3 | 0.00011 |
| E-Jka- | 125 | 55 | 0.00204 | E-Fyb-Jka-K-s- | 5 | 3 | 0.00011 |
| E-Jkb-K-S- | 112 | 55 | 0.00204 | E-Jka-s- | 3 | 3 | 0.00011 |
| c-E-K-M- | 118 | 54 | 0.002 | Fya-M- | 10 | 3 | 0.00011 |
| Fyb-K- | 131 | 54 | 0.002 | Jka-Jkb- | 4 | 3 | 0.00011 |
| c-E-K-S- | 116 | 51 | 0.00189 | Jka-Jkb-K- | 4 | 3 | 0.00011 |
| C- | 152 | 49 | 0.00181 | Jka-K-M- | 3 | 3 | 0.00011 |
| C-e-Fyb-K- | 84 | 49 | 0.00181 | c-E-Fya-Jka- | 2 | 2 | 0.00007 |
| c-E-Jkb-K-S- | 99 | 48 | 0.00178 | C-e-Fya-Jkb- | 3 | 2 | 0.00007 |
| C-E-Fyb-K- | 87 | 46 | 0.0017 | c-E-Fya-K-M- | 5 | 2 | 0.00007 |
| C-E-Fya-Jka-K- | 130 | 44 | 0.00163 | c-E-Fya-M-S- | 2 | 2 | 0.00007 |
| E-Fya-Jkb-K-S- | 89 | 44 | 0.00163 | C-E-Fya-S- | 6 | 2 | 0.00007 |
| C-e-Fya-K- | 92 | 43 | 0.00159 | C-e-Fyb-Jka-K- | 3 | 2 | 0.00007 |
| E-Fya- | 100 | 40 | 0.00148 | c-E-Fyb-K-M- | 9 | 2 | 0.00007 |
| C-Fya-K-S- | 94 | 39 | 0.00144 | c-E-Jka-K-M-S- | 2 | 2 | 0.00007 |
| C-K-S- | 92 | 39 | 0.00144 | c-E-Jka-K-s- | 6 | 2 | 0.00007 |
| C-E-Jkb-K-S- | 64 | 38 | 0.00141 | C-E-Jkb-S- | 4 | 2 | 0.00007 |
| c-E-Jka-K-S- | 72 | 37 | 0.00137 | C-e-K-M-S- | 12 | 2 | 0.00007 |
| e- | 118 | 36 | 0.00133 | C-e-K-s- | 6 | 2 | 0.00007 |
| c-E-Fyb-K-S- | 69 | 35 | 0.0013 | c-E-s- | 4 | 2 | 0.00007 |
| c-E-Fya-Jka-K- | 79 | 34 | 0.00126 | c-Fya- | 4 | 2 | 0.00007 |
| c-E-Fya-Jkb-K-S- | 60 | 33 | 0.00122 | C-Fya-Jka-S- | 3 | 2 | 0.00007 |
| C-E-K-M- | 73 | 33 | 0.00122 | C-Fya-S- | 6 | 2 | 0.00007 |
| c-E-Jka- | 66 | 32 | 0.00119 | c-Fyb-Jka-K- | 2 | 2 | 0.00007 |
| E-Fya-K-s- | 49 | 32 | 0.00119 | C-Fyb-Jka-K-M-S- | 4 | 2 | 0.00007 |
| E-Fyb-Jkb-K-S- | 49 | 31 | 0.00115 | C-Fyb-Jkb-K-S- | 4 | 2 | 0.00007 |
| E-K-s- | 69 | 31 | 0.00115 | C-Fyb-K-S- | 4 | 2 | 0.00007 |
| Jkb- | 86 | 28 | 0.00104 | c-Jka- | 5 | 2 | 0.00007 |
| C-E-Fyb-Jkb-K- | 67 | 27 | 0.001 | C-Jka-K-s- | 3 | 2 | 0.00007 |
| C-e-Jka- | 57 | 27 | 0.001 | c-Jkb-K- | 4 | 2 | 0.00007 |
| E-Fya-Jka-K-S- | 46 | 26 | 0.00096 | C-k- | 2 | 2 | 0.00007 |
| E-Fyb-Jka-K-S- | 49 | 26 | 0.00096 | C-K-M-S- | 8 | 2 | 0.00007 |
| E-Fyb-Jka-K- | 42 | 25 | 0.00093 | C-M- | 3 | 2 | 0.00007 |
| E-Jkb- | 58 | 25 | 0.00093 | E-Fyb-K-M- | 6 | 2 | 0.00007 |
| C-e-Jkb-K- | 42 | 23 | 0.00085 | E-Jkb-s- | 2 | 2 | 0.00007 |
| Fya-Jka-K- | 50 | 23 | 0.00085 | e-K-M- | 4 | 2 | 0.00007 |
| S- | 58 | 23 | 0.00085 | E-M-S- | 5 | 2 | 0.00007 |
| E-Fya-K-M-S- | 39 | 22 | 0.00081 | Fya-Jka-K-S- | 4 | 2 | 0.00007 |
| c-E-Fya- | 53 | 21 | 0.00078 | Fyb-Jkb-K-s- | 4 | 2 | 0.00007 |
| Fyb-Jka- | 38 | 21 | 0.00078 | Fyb-Jkb-K-S- | 5 | 2 | 0.00007 |
| C-Fyb-Jka-K- | 32 | 20 | 0.00074 | Fyb-K-M- | 3 | 2 | 0.00007 |
| C-e-Fya- | 59 | 19 | 0.0007 | Jka-s- | 2 | 2 | 0.00007 |
| C-Fya-Jka-K- | 26 | 19 | 0.0007 | Jkb-k- | 2 | 2 | 0.00007 |
| E-Fyb-K-S- | 40 | 19 | 0.0007 | Jkb-K-s- | 3 | 2 | 0.00007 |
| Fya-Jka- | 54 | 19 | 0.0007 | Jkb-s- | 2 | 2 | 0.00007 |
| K-s- | 34 | 19 | 0.0007 | s- | 6 | 2 | 0.00007 |
| C-e-K-M- | 29 | 18 | 0.00067 | C-E-Fya-Fyb-Jka-K- | 4 | 1 | 0.00004 |
| C-Jkb-K-s- | 33 | 18 | 0.00067 | C-E-Fya-Jka-K-M- | 1 | 1 | 0.00004 |
| C-E-Jkb- | 43 | 17 | 0.00063 | C-E-Fya-Jka-K-s- | 1 | 1 | 0.00004 |
| C-K-M- | 27 | 17 | 0.00063 | C-E-Fya-Jkb- | 1 | 1 | 0.00004 |
| C-E-Fyb-Jkb-K-S- | 28 | 16 | 0.00059 | c-E-Fya-Jkb-K-M-S- | 4 | 1 | 0.00004 |
| c-E-K-M-S- | 31 | 16 | 0.00059 | c-E-Fya-Jkb-S- | 4 | 1 | 0.00004 |
| Jkb-K-S- | 29 | 16 | 0.00059 | c-E-Fya-s- | 4 | 1 | 0.00004 |
| C-E-Fyb-Jka-K- | 24 | 15 | 0.00056 | C-E-Fya-s- | 1 | 1 | 0.00004 |
| C-Jka- | 37 | 15 | 0.00056 | C-E-Fyb-Jka-K-s- | 1 | 1 | 0.00004 |
| Fyb- | 35 | 15 | 0.00056 | c-E-Fyb-Jka-S- | 2 | 1 | 0.00004 |
| c-E-Fya-Jka-K-S- | 23 | 14 | 0.00052 | C-E-Fyb-Jka-S- | 2 | 1 | 0.00004 |
| c-E-Fyb-Jkb-K-S- | 24 | 14 | 0.00052 | c-E-Fyb-Jkb- | 3 | 1 | 0.00004 |
| e-K- | 35 | 14 | 0.00052 | C-e-Fyb-K-S- | 1 | 1 | 0.00004 |
| E-k- | 17 | 14 | 0.00052 | c-E-Jka-Jkb-K- | 1 | 1 | 0.00004 |
| K-M-S- | 38 | 14 | 0.00052 | C-E-Jka-K-M-S- | 3 | 1 | 0.00004 |
| c-E-Fyb-Jka-K-S- | 18 | 13 | 0.00048 | C-e-Jka-K-s- | 1 | 1 | 0.00004 |
| c-E-S- | 40 | 13 | 0.00048 | c-E-Jka-S- | 2 | 1 | 0.00004 |
| C-E-Fya- | 31 | 12 | 0.00044 | C-E-Jka-S- | 1 | 1 | 0.00004 |
| C-E-Jka-K-S- | 25 | 12 | 0.00044 | c-E-Jkb-S- | 2 | 1 | 0.00004 |
| C-e-K-S- | 25 | 12 | 0.00044 | C-E-K-Jka- | 3 | 1 | 0.00004 |
| C-Fyb-K- | 35 | 12 | 0.00044 | c-E-k-S- | 2 | 1 | 0.00004 |
| E-Fya-K-M- | 23 | 12 | 0.00044 | C-E-K-s- | 2 | 1 | 0.00004 |
| E-S- | 31 | 12 | 0.00044 | C-e-M- | 2 | 1 | 0.00004 |
| Fya-Jkb-K-s- | 21 | 12 | 0.00044 | C-E-M- | 2 | 1 | 0.00004 |
| Fyb-K-S- | 31 | 12 | 0.00044 | C-E-M-S- | 2 | 1 | 0.00004 |
| c-E-Jkb- | 27 | 11 | 0.00041 | C-Fya-Jka-M-S- | 1 | 1 | 0.00004 |
| c-Jkb- | 23 | 11 | 0.00041 | c-Fya-Jkb-K- | 2 | 1 | 0.00004 |
| E-Jkb-K-s- | 18 | 11 | 0.00041 | C-Fya-Jkb-K-S- | 3 | 1 | 0.00004 |
| E-K-M-S- | 23 | 11 | 0.00041 | c-Fya-K-S- | 2 | 1 | 0.00004 |
| k- | 18 | 11 | 0.00041 | C-Fyb- | 2 | 1 | 0.00004 |
| Fya-K-S- | 23 | 10 | 0.00037 | C-Fyb-Jka-K-S- | 1 | 1 | 0.00004 |
| c-E-Fya-Jkb- | 13 | 8 | 0.0003 | c-Jka-M- | 2 | 1 | 0.00004 |
| c-E-Fya-K-s- | 16 | 8 | 0.0003 | C-Jka-M- | 2 | 1 | 0.00004 |
| C-E-Jka- | 15 | 8 | 0.0003 | C-Jkb- | 4 | 1 | 0.00004 |
| C-E-Jkb-K-M- | 22 | 8 | 0.0003 | c-Jkb-K-S- | 4 | 1 | 0.00004 |
| C-e-S- | 14 | 8 | 0.0003 | c-K-S- | 2 | 1 | 0.00004 |
| C-Fya- | 18 | 8 | 0.0003 | c-S- | 3 | 1 | 0.00004 |
| C-Fya-K-s- | 13 | 8 | 0.0003 | C-s- | 2 | 1 | 0.00004 |
| E-Fya-S- | 24 | 8 | 0.0003 | E-Fyb-Jkb-S- | 1 | 1 | 0.00004 |
| E-Fyb-Jka- | 12 | 8 | 0.0003 | E-Fyb-K-M-S- | 3 | 1 | 0.00004 |
| E-Jka-K-M- | 16 | 8 | 0.0003 | E-Jka-K-s- | 1 | 1 | 0.00004 |
| Fya-Jkb- | 17 | 8 | 0.0003 | E-Jka-M- | 2 | 1 | 0.00004 |
| Jka-S- | 19 | 8 | 0.0003 | E-Jkb-K-M-S- | 2 | 1 | 0.00004 |
| C-E-Fya-Jka-K-M-S- | 7 | 7 | 0.00026 | E-Jkb-S- | 2 | 1 | 0.00004 |
| C-e-Fya-Jkb-K- | 18 | 7 | 0.00026 | e-K-M-S- | 1 | 1 | 0.00004 |
| c-E-Fya-Jkb-K-s- | 8 | 7 | 0.00026 | Fya-Jka-K-M- | 1 | 1 | 0.00004 |
| c-E-Fya-K-M-S- | 11 | 7 | 0.00026 | Fya-Jkb-K-M-S- | 1 | 1 | 0.00004 |
| C-e-Fya-K-S- | 13 | 7 | 0.00026 | Fya-K-s- | 1 | 1 | 0.00004 |
| c-E-Fya-S- | 14 | 7 | 0.00026 | Fyb-Jka-K-s- | 3 | 1 | 0.00004 |
| C-E-Jkb-K-s- | 13 | 7 | 0.00026 | Fyb-Jka-K-S- | 4 | 1 | 0.00004 |
| C-E-K-M-S- | 17 | 7 | 0.00026 | Fyb-Jkb-K-M- | 1 | 1 | 0.00004 |
| E-Fyb-K-s- | 12 | 7 | 0.00026 | Fyb-S- | 2 | 1 | 0.00004 |
| Fya-S- | 17 | 7 | 0.00026 | Jka-K-M-S- | 2 | 1 | 0.00004 |
| c- | 26 | 6 | 0.00022 |  |  |  |  |
