# Supplementary material for: Probabilistic mathematical modelling to predict the red cell phenotyped donor panel size
Source: PLoS One. 2022 Nov 11;17(11):e0276780. doi: 10.1371/journal.pone.0276780 (PMC9651592; doi:10.1371/journal.pone.0276780)
Supplement: Attachment — Submitted filename: PONE-D-21-20968_reviewer.pdf [file pone.0276780.s002.pdf]

PLOS ONE

Probabilistic mathematical modelling to predict the red cell phenotyped donor panel size

--Manuscript Draft--

**Manuscript Number:**	PONE-D-21-20968
**Article Type:**	Research Article
**Full Title:**	Probabilistic mathematical modelling to predict the red cell phenotyped donor panel size
**Short Title:**	Mathematical modelling for blood donor panel
**Corresponding Author:**	Shamila Ginige<br>Australian Red Cross Lifeblood<br>Kelvin Grove, AUSTRALIA
**Keywords:**	Mathematical model; probabilities; genotyping; phenotyping; donor panel; probabilistic model; blood group
**Abstract:**	Background: In the last decade, Australia has experienced an overall decline in red cell demand, but there has been an increased need for phenotyped matched red cells. Lifeblood and mathematicians from Queensland universities have developed a probabilistic model to determine the percentage of the donor panel that would need extended antigen typing to meet this increasing demand, and an estimated timeframe to achieve the optimum required phenotyped (genotyped) panel.<br>Methods: Mathematical modelling, based on Multinomial distributions, was used to provide guidance on the percentage of typed donor panel needed, based on recent historical blood request data and the current donor panel size. Only antigen combinations determined to be uncommon, but not rare, were considered. Simulations were run to attain a 95% confidence interval.<br>Results: Modelling predicted a target of 38% of the donor panel, or 205,000 donors, would need to be genotyped to meet the current demand. If 5% of weekly returning donors were genotyped, this target would be reached within 12 years. For phenotyping, 35% or 188,000 donors would need to be phenotyped to meet Lifeblood's demand. With the current level of testing, this would take eight years but could be performed within three years if testing was increased to 9% of weekly returning donors. An additional 26,140 returning donors need to be phenotyped annually to maintain this panel.<br>Conclusion: This mathematical model will inform business decisions and assist Lifeblood in determining the level of investment required to meet the desired timeframe to achieve the optimum donor panel size.
**Order of Authors:**	Shamila Ginige
	Kevin Burrage
	Pamela Burrage
	Diane Donovan
	Bevan Thompson
	Tanya Powley
	Denisse Best
	James Daly
**Additional Information:**	
**Question**	**Response**
**Financial Disclosure**<br><br>Enter a financial disclosure statement that describes the sources of funding for the	The author(s) received no specific funding for this work.

work included in this submission. Review the submission guidelines for detailed requirements. View published research articles from *PLOS ONE* for specific examples.<br><br>This statement is required for submission and **will appear in the published article** if the submission is accepted. Please make sure it is accurate.<br><br>**Unfunded studies**<br>Enter: *The author(s) received no specific funding for this work.*<br><br>**Funded studies**<br>Enter a statement with the following details:<br>• Initials of the authors who received each award<br>• Grant numbers awarded to each author<br>• The full name of each funder<br>• URL of each funder website<br>• Did the sponsors or funders play any role in the study design, data collection and analysis, decision to publish, or preparation of the manuscript?<br>• **NO** - Include this sentence at the end of your statement: *The funders had no role in study design, data collection and analysis, decision to publish, or preparation of the manuscript.*<br>• **YES** - Specify the role(s) played.<br><br>* typeset	
**Competing Interests**<br><br>Use the instructions below to enter a competing interest statement for this submission. On behalf of all authors, disclose any competing interests that could be perceived to bias this work—acknowledging all financial support and any other relevant financial or non-financial competing interests.<br><br>This statement is required for submission and **will appear in the published article** if the submission is accepted. Please make sure it is accurate and that any funding sources listed in your Funding Information	The authors have declared that no competing interests exist.

later in the submission form are also declared in your Financial Disclosure statement.<br><br>View published research articles from *PLOS ONE* for specific examples.<br><br>**NO authors have competing interests**<br><br>Enter: *The authors have declared that no competing interests exist.*<br><br>**Authors with competing interests**<br><br>Enter competing interest details beginning with this statement:<br><br>*I have read the journal's policy and the authors of this manuscript have the following competing interests: [insert competing interests here]*<br><br>* typeset	
**Ethics Statement**<br><br>Enter an ethics statement for this submission. This statement is required if the study involved:<br><br>• Human participants<br>• Human specimens or tissue<br>• Vertebrate animals or cephalopods<br>• Vertebrate embryos or tissues<br>• Field research<br><br>Write "N/A" if the submission does not require an ethics statement.<br><br>General guidance is provided below. Consult the submission guidelines for detailed instructions. **Make sure that all information entered here is included in the Methods section of the manuscript.**	N/A

**Format for specific study types**<br><br>**Human Subject Research (involving human participants and/or tissue)**<br>• Give the name of the institutional review board or ethics committee that approved the study<br>• Include the approval number and/or a statement indicating approval of this research<br>• Indicate the form of consent obtained (written/oral) or the reason that consent was not obtained (e.g. the data were analyzed anonymously)<br><br>**Animal Research (involving vertebrate animals, embryos or tissues)**<br>• Provide the name of the Institutional Animal Care and Use Committee (IACUC) or other relevant ethics board that reviewed the study protocol, and indicate whether they approved this research or granted a formal waiver of ethical approval<br>• Include an approval number if one was obtained<br>• If the study involved *non-human primates*, add *additional details* about animal welfare and steps taken to ameliorate suffering<br>• If anesthesia, euthanasia, or any kind of animal sacrifice is part of the study, include briefly which substances and/or methods were applied<br><br>**Field Research**<br><br>Include the following details if this study involves the collection of plant, animal, or other materials from a natural setting:<br>• Field permit number<br>• Name of the institution or relevant body that granted permission	
**Data Availability**<br><br>Authors are required to make all data underlying the findings described fully available, without restriction, and from the time of publication. PLOS allows rare exceptions to address legal and ethical concerns. See the PLOS Data Policy and FAQ for detailed information.	Yes - all data are fully available without restriction

A Data Availability Statement describing where the data can be found is required at submission. Your answers to this question constitute the Data Availability Statement and **will be published in the article**, if accepted.<br><br>**Important:** Stating 'data available on request from the author' is not sufficient. If your data are only available upon request, select 'No' for the first question and explain your exceptional situation in the text box.<br><br>Do the authors confirm that all data underlying the findings described in their manuscript are fully available without restriction?	
**Describe where the data may be found in full sentences. If you are copying our sample text, replace any instances of XXX with the appropriate details.**<br><br>• If the data are **held or will be held in a public repository**, include URLs, accession numbers or DOIs. If this information will only be available after acceptance, indicate this by ticking the box below. For example: *All XXX files are available from the XXX database (accession number(s) XXX, XXX.)*.<br>• If the data are all contained **within the manuscript and/or Supporting Information files**, enter the following: *All relevant data are within the manuscript and its Supporting Information files.*<br>• If neither of these applies but you are able to provide **details of access elsewhere**, with or without limitations, please do so. For example:<br><br>*Data cannot be shared publicly because of [XXX]. Data are available from the XXX Institutional Data Access / Ethics Committee (contact via XXX) for researchers who meet the criteria for access to confidential data.*<br><br>*The data underlying the results presented in the study are available from (include the name of the third party*	All relevant data are within the manuscript and its Supporting Information files.

and contact information or URL).<br>• This text is appropriate if the data are owned by a third party and authors do not have permission to share the data.<br><br>* typeset	
Additional data availability information:	

Manuscript Click here to access/download;Manuscript;Main text file.docx

Title: Probabilistic mathematical modelling to predict the red cell phenotyped donor panel size

Short title: Mathematical modelling for blood donor panel

Authors: Shamila Ginige[1] Kevin Burrage[2], Pamela Burrage[2], Diane Donovan[3], Bevan Thompson[3], Tanya Powley[1], Denisse Best[1], James Daly[1]

Affiliations: [1]Australian Red Cross Lifeblood, [2]School of Mathematical Sciences, Queensland University of Technology, [3]School of Mathematics and Physics, University of Queensland

Address correspondence to: Brisbane Processing Centre, Australian Red Cross Lifeblood. PO Box 1197, Stafford, QLD 4053, +61 7 3838 9039, Fax: +61 7 3838 9421, Email: dbest@redcrossblood.org.au

Word Count: 4,339 words

Abstract

Background: In the last decade, Australia has experienced an overall decline in red cell demand, but there has been an increased need for phenotyped matched red cells. Lifeblood and mathematicians from Queensland universities have developed a probabilistic model to determine the percentage of the donor panel that would need extended antigen typing to meet this increasing demand, and an estimated timeframe to achieve the optimum required phenotyped (genotyped) panel.

Methods: Mathematical modelling, based on Multinomial distributions, was used to provide guidance on the percentage of typed donor panel needed, based on recent historical blood request data and the current donor panel size. Only antigen combinations determined to be uncommon, but not rare, were considered. Simulations were run to attain a 95% confidence interval.

Results: Modelling predicted a target of 38% of the donor panel, or 205,000 donors, would need to be genotyped to meet the current demand. If 5% of weekly returning donors were genotyped, this target would be reached within 12 years. For phenotyping, 35% or 188,000 donors would need to be phenotyped to meet Lifeblood’s demand. With the current level of testing, this would take eight years but could be performed within three years if testing was increased to 9% of weekly returning donors. An additional 26,140 returning donors need to be phenotyped annually to maintain this panel.

Conclusion: This mathematical model will inform business decisions and assist Lifeblood in determining the level of investment required to meet the desired timeframe to achieve the optimum donor panel size.

Keywords

Mathematical model
Probabilities
Genotyping
Phenotyping
Donor panel

Introduction

The International Society for Blood Transfusion Committee on Terminology for Red Cell Surface Antigens has recognized over 300 blood group antigens. The combinations of these blood group antigens varies between individuals so transfused red blood cells (RBC) have the potential to cause an immune response in recipients that lack certain antigens. Patients who have red cell antibodies that are considered clinically significant will require red cells that are negative for the corresponding antigen. Depending on the specific alloantibody in the recipient and the prevalence of the blood group antigen in a donor population, delivery of compatible RBC can be difficult [1]. Patients that have specific conditions or are on treatments that make red cell antibodies difficult to exclude, or where it is considered beneficial to reduce the risk of alloimmunisation may also require transfusion with red cells lacking one or more blood group antigens. The limited shelf life of red cell products and the reliance of blood donor appointments makes the estimation of the phenotyped inventory complex and challenging.

While there has been an overall reduction in the demand for RBC in Australia (approximately 20% since 2011/12), there has been a rise in the demand for phenotype matched red cells (approximately 50% over three years). As the sole provider of fresh blood components to the Australian population, the Australian Red Cross Lifeblood has a keen interest in identifying blood donors with a range of red cell phenotypes.

Lifeblood routinely performs ABO, Rh (D, C, E, c and e) and K typing to match blood donors and recipients. Extended antigen typing with antibody-based serological methods can be laborious, expensive, and limited to reagent antibody availability and is only performed on a proportion of repeat donors. When reagent antibodies are not available for phenotyping, DNA microarrays that target specific single nucleotide polymorphisms (SNPs) can also be used for extended blood group typing [3]. Blood donors who have been genotyped on previous donations will have historical genotype information that can predict the phenotype and be used without requiring re-testing [2].

Mass screening of blood donors with extended phenotyping and genotyping provides the opportunity to create and maintain a large database to meet demand for specific antigen-negative requirements. The additional cost of extended blood group

phenotyping and/or genotyping generally precludes testing the entire blood donor panel. However, there is limited literature on the optimum percentage of the donor pool that is required to be tested to reliably meet the demand for phenotype matched red cells.

The majority of molecular blood group screening programs should be able to cover around 97% of all clinically important blood groups [4]. Portegysa et al. [5] describes one such regional donor registry established in Germany that provides matched blood products on demand.

The Canadian Blood Service (CBS) is responsible for providing blood to all areas of Canada, outside of Quebec, and has approximately 450,000 donors providing around 900,000 units of RBCs annually. It was estimated that 20-30% of these donors have some level of extended phenotyping, beginning with extended Rh (C, c, E and e) and K phenotyping and then on to Duffy (Fy^a and Fy^b), Kidd (Jk^a and Jk^b), S and s typing on subsequent donations if indicated [6]. CBS found that with a screening rate of 30%, it would take three to five years to achieve a sufficient registry to service their population [6]. Following on from this, they estimated an ongoing screening rate of 10-20% for their new donors to maintain a stable inventory. This analysis was based on a rare donor turnover of less than 10% each year, and the addition of related donors for each new rare donor discovered from the screening [6].

Similarly, Hema-Quebec implemented a strategy to create a database from genotyping 21,000 frequent blood donors over two years [7]. Their analysis demonstrated that this database should provide enough variation in blood group antigens to cover 95% of the 16,500 annual hospital requests for phenotyped RBCs. They also estimated that they would need to genotype 4,000 new donors each year to maintain this database.

The Bloodcenter of Wisconsin in the United States of America (USA) performed mass-scale genotyping on their blood donors over four years and by the final year of the program, 29% of all blood units on any given day had a known genotype. This allowed 99.8% of patient encounters to be resolved by their own database and inventory. The Bloodcenter of Wisconsin determined that each year, 4,000 donors would need to be genotyped to maintain this database [2] [8].

In the Netherlands, the Sanquin Bank of Frozen Blood (SBFB) runs a national typing program of blood donors. All donors are typed for ABO, Rh and K typing, with K positive donors being typed for k. In addition, 30% of donors were further typed for Fy^a, Fy^b, Jk^a, Jk^b, M, N, S and s and a further few were typed for Kp^a, C^w, Co^b, Wr^a, Lu^a, Le^a, Le^b and P1. With this database, Sanquin were able to find compatible units in 95% of requested cases [9].

In collaboration with mathematicians from Queensland universities, Lifeblood conducted this project to develop a mathematical model to determine:

- the percentage of the Australian whole blood donor panel requiring additional phenotyping or genotyping, to ensure that sufficient donors with the commonly ordered antigen combinations are available to meet demand;
- the timescale for achieving the minimum percentage of phenotyped or genotyped donors;
- how this timescale may be reduced by increasing the percentage of inventory tested;
- the percentage of inventory to be tested to maintain the optimum number of whole blood donor phenotyped once the target has been reached.

There is very little modelling work in the literature to address this or similar blood demand problems but we highlight work done by Blake and Clarke [6]. In their paper a two-phase approach was developed to determine how rare a blood type needed to be before freezing and the associated screening rates. Discrete event simulations were run but their model only evaluated a single antigen at a time. When exploring scenarios based around 29 different antigens, these were treated independently, so that the underlying statistical distributions are essentially Binomial.

In our modelling approach we will not assume independence, as we now explain. A natural modelling approach is based around the Multinomial distribution, where probabilities are associated with a list of “antigen combinations” based on the ABO blood group system, as well as a combination of 40 other blood group systems and over 300 different blood group antigens.

Methods (2,755 words)

To estimate the level of phenotyping and genotyping to meet clinical demand with a 95% confidence level, we started with a set of individuals (donors) to be allocated (typed) across *r* disjoint independent categories (antigen combinations). Each category was assigned a target (number of donors) and a probability of assigning an individual to that category. Then we predicted the number of individuals (donors) required to reach the targets under a random allocation, refer to Figure 1.

Figure 1: A) Donor panel and indicative probability distribution for r categories (shown as blood bags); B) Given the probabilities for each category, donors are assigned at random to the categories.

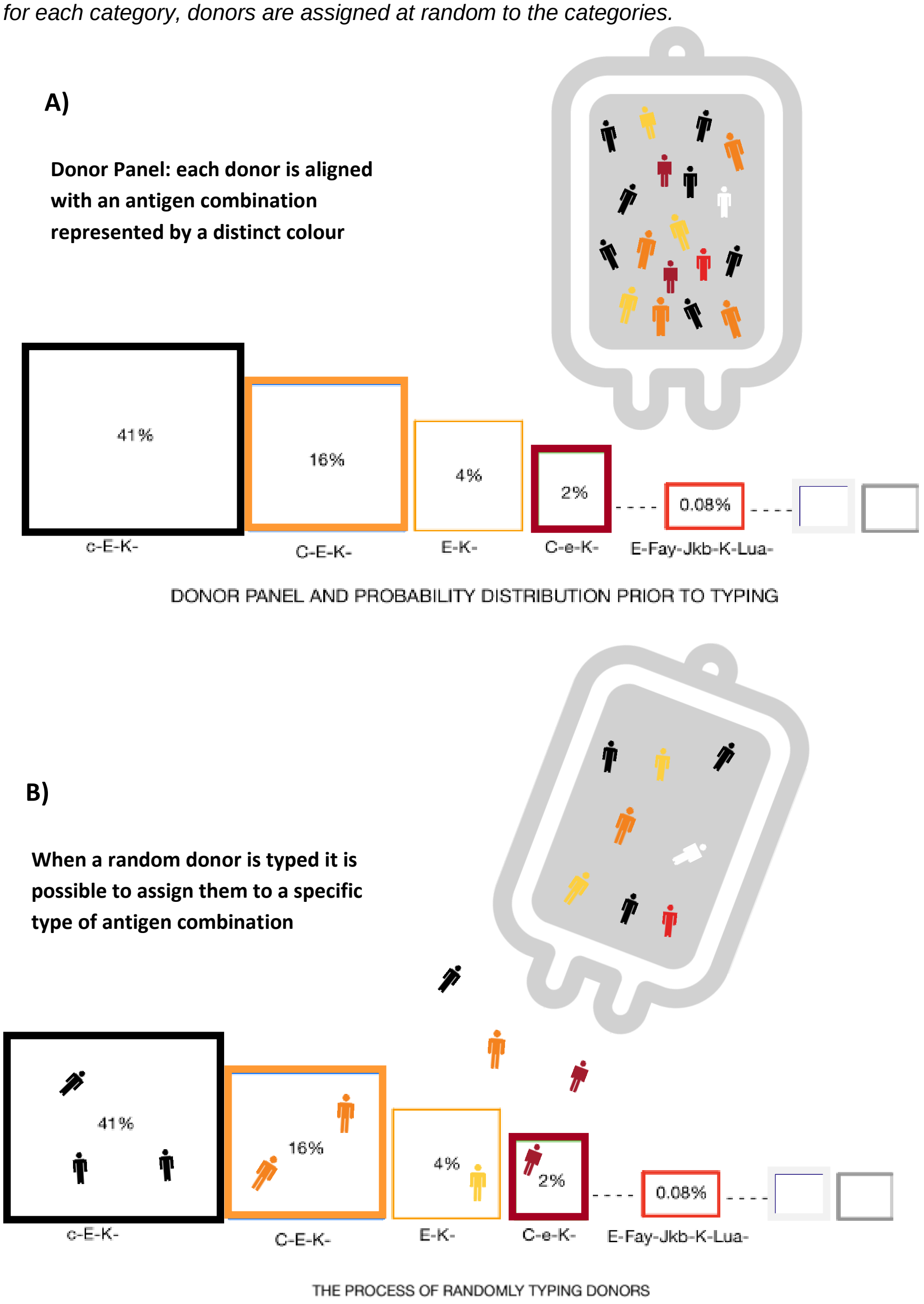

A crucial step in this process was the determination of the probability distribution. Whilst each antigen has its own probability distribution, these are generally population specific and the occurrence of each antigen in a particular antigen combination is not always independent. For example, the probability of a phenotype E-, Fy[a]-, Jk[b]-, K- and Lu[a]- may not be the product of the probabilities of the individual components. To address this issue, we used historical frequency information from previous Lifeblood requests to build a set of probabilities for each of the categories (combination of antigens) requested. The probabilities of the antigen combinations of interest were calculat[illegible] by taking the frequency of a requested product (antigen combination) as a percentage of the total number of requests, refer to Figure 2. It was assumed that the historical request data, used to set targets for the allocation of donors to *r* categories, was sufficiently comprehensive to determine reasonable estimates for the probabilities of each of these categories.

Each donor is assumed to be aligned with a unique category; the allocation to a category is achieved by a random process based on a Multinomial distribution where *S* donors are ~~sequentially~~ allocated across *r* categories based on the category probabilities in Figure 2. If after *S* donors are allocated, the targets for all the categories are not reached, then the number of donors is increased until all the targets are met. To ensure the targets for the uncommon phenotypes are met, the process is continued until at least one donor is allocated to each category[illegible]his random process ensures that more donors are allocated to categories with higher probabilities, meeting these targets as well. In building the Multinomial distribution model we used the following framework:

Under a Multinomial distribution on the category types $A_1, \cdots, A_r$, with probabilities $p_1, \cdots, p_r \in [0,1]$ where $p_1 + \cdots + p_r = 1$, the probability of obtaining typed donors to match $(n_1, \cdots, n_r)$ requests, where $\sum_{i=1}^{r} n_i = S$, is given by

$$P = Prob(n_1, \cdots, n_r)$$

$$= \frac{S!}{\prod_{j=1}^{r} n_j!} \prod_{j=1}^{r} p_j^{n_j}.$$

Here $\prod_{j=1}^{r} p_j^{n_j}$ denotes the product of *r* terms, $p_j^{n_j}$ over $j = 1, \cdots, r,$ and $a! = a.(a-1).(a-2) \cdots 2.1.$

Table 1: Notation to be used in the specification of the Multinomial distribution

Glossary	
Notation	Specification
S	Number of donors to be typed
r	Number of distinct categories (unique identifies, products)
$A = (A_1, \cdots, A_r)$	Vector containing the distinct categories
$Nvec = (n_1, \cdots, n_r)$	Vector containing the number of typed donors in each category
$Pvec = (p_1, \cdots, p_r)$	The vector of probabilities, one for each category
$Kvec = (k_1, \cdots, k_r)$	Targets for the number of typed donors for each category

The following theorem underpins the mathematical model:

Theorem 1: Given the above Multinomial distribution where *S* donors are to be allocated to *r* categories, $A_1, \cdots, A_r$, with probabilities $p_1, \cdots, p_r$ then the expected number of donors to be allocated to category A_i is given by $\mathbb{E}[n_i]$, with variance $\mathbb{V}ar[n_i]$, where

$$\mathbb{E}[n_i] = p_i S, \quad \mathbb{V}ar[n_i] = p_i(1 - p_i)S, \quad i = 1, \cdots, r;$$

noting that the marginal distributions are Binomial distributions.

The vector of given targets (*Kvec*) and the sum of these targets (*K*), are used to estimate the size of *S* so that all the components of the vector of targets are met with a certain probability. The size *S* must be greater than or equal to *K*. However, it is very unlikely to be equal to *K* as some of the Multinomial samples needed to match a given component of the target vector may be considerably greater than the individual targets. We estimated *S* and the associated probability.

$$P_S = Prob(n_1 \geq k_1, \cdots, n_r \geq k_r)$$

$$\text{where} \quad \sum_{j=1}^{r} n_j = S, \; n_j \geq k_j \text{ for } j = 1, \cdots, r.$$

Intuitively if $S - K$ is positive but close to 0, we would expect the probability P_S to be very close to 0. However as $S - K$ becomes larger, the probability of meeting the targets approaches 1.

One way of addressing this problem is to perform a number of Multinomial trials to estimate *S* for a given set of targets and probabilities. Unfortunately, this is not a

computationally feasible approach even for moderate sizes of *r*. However, when *S* is large, the following theorem can be applied to find good approximations to these probabilities and good estimates of *S*.

Theorem 2: The following lower and upper bounds on P_S hold

$$1-\sum_{j=1}^{r} Prob(\,n_j < k_j) \;\leq P_S \leq \prod_{j=1}^{r} Prob\big(n_j \geq k_j\big) \;\leq exp\left(-\sum Prob\big(n_j < k_j\big)\right).$$

Proof: Mallows [10].

Here the individual probabilities $Prob(n_j < k_j)$ and $Prob\big(n_j \geq k_j\big)$ are the marginal (binomial) probabilities and are very quick to compute. Importantly, as *S* becomes large and the probability P_S gets closer to 1 then these bounds get close together, and so provide an excellent approximation to the probability P_S . We chose a high probability P_S of 0.95.

The sample size S_{95} was determined with the lower bound being greater than 0.95

$$1-\sum_{j=1}^{r} Prob(\,n_j < k_j) > 0.95, \text{ where}$$

$$\sum_{j=1}^{r} n_j = S_{95}, \qquad n_j \geq k_j \text{ for } j = 1,\cdots,r.$$

Once we have estimated *S* in this manner, we validate the results by running a set of Monte Carlo Multinomial simulations to determine if indicative request targets can be met within given time periods.

For a given *S* we simulate from the appropriate Multinomial distribution and record the vector $Nvec = (n_1,\cdots,n_r)$ as the number of donors allocated to each category. The number of negative terms in the difference $Nvec - Kvec = (n_1 - k_1,\cdots,n_r - k_r)$ represents the number of times a target is not attained.

We ran 10,000 Monte Carlo Multinomial simulations and computed the number of times that $Nvec - Kvec$ has a negative component in any position. The number of failures to meet targets was calculated as a percentage of the number of categories *r*. Taking one hundred minus this value gave the minimum percentage of success in attaining the

targets. The Monte Carlo Multinomial simulations were repeated using increasing values of S until a 95% confidence level was obtained. This was compared with the estimates computed by the approach of Mallows, described in Theorem 2.

Determination of testing regimes needed to meet targets within given timeframes

Using the described mathematical framework, formulae were developed to estimate the timeframes needed to meet the required targets. These estimates were based on bounds given in Theorem 2.

Table 2: Notation for calculation of timelines

Glossary	
Notation	Specification
D_0	Number of donors already screened
D_y	Number of donors screened at end of year *y*
q	The number donors screened per annum
m	The percentage of non-returning screened donors per annum as a decimal

Using the notation in Table 2, the number of screened donors at the end of year 1 is

$$D_1 = (1-m)D_0 + q(1-m/$$ [illegible]

Setting $M = q(1 - m/2)$, the number of screened donors at the end of year 2 is

$$D_2 = (1-m)\big((1-m)D_0 + M\big) + M \; = \; (1-m)^2 D_0 + M(1-m) + M.$$

By induction, the number of screened donors at the end of *y* years is

$$D_y = \; (1-m)^y D_0 + M((1-m)^{y-1} + (1-m)^{y-2} + \cdots + (1-m)^1 + 1)$$

$$= (1-m)^y D_0 + \frac{M}{m}(1-(1-m)^y).$$

This formula enabled calculation of either *q* or the number of years *y*, given knowledge of the targeted number of donors D_y and the initial number of donors already screened D_0.

Results

The mathematical model was used to analyse supply and demand data for phenotyped red cells. Lifeblood's phenotyped red cell demand for approximately 27000 requests was analysed. The requests for antigen negative combinations achieved through extended phenotyping by serology or genotyping to predict the phenotype for the following antigens:

Serology: C, c, E, e, K, k, Fy^a, Fy^b, Jk^a, Jk^b, M, S and s

Genotyping: C, c, E, e, VS, V, K, k, Js^a, Js^b, Kp^a, Kp^b, Fy^a, Fy^b, Jk^a, Jk^b, M, N, S, s, U, Lu^a, Lu^b, Do^a, Do^b, Hy, Jo^a, LW^a, LW^b, Di^a, Di^b, Co^a, Co^b, Sc1 and Sc2

Examples of the antigen combinations can be found in Table 4, where a full description of all 603 antigen combinations is provided as a supplement.

The current testing technology allows for red cell products to be categorised as one of 403 distinct antigen combinations using genotyping, and 299 distinct antigen combinations using extended phenotyping. For this study the following assumptions were used:

- donor panel consisted of 542,891 donors
- approximately 700,000 donations collected annually
- 13% of new donors did not return for a second donation
- 17% of the panel is replaced with new donors each year
- donors donate no more than 3 times per year
- 12% of the donor panel has already been phenotyped
- 670 donors are phenotyped each week
- 135 donors are genotyped each week

The probability for each antigen combination was determined, and are provided in the supplement. The probabilities for the 403 genotype antigen combinations are displayed in Figure 2 and Figure 3. The antigen combination with the highest frequency of being requested is c-E-K- with a probability of 0.41. Figure 2 emphasises the fact that the

probabilities decay rapidly. To provide better resolution, the probabilities have been replotted in Figure 3 with the higher probability antigen combinations suppressed. It can be seen that approximately 93% of the antigen combinations have a frequency of less than 4 in 1000. It is acknowledged that, in practice, requests for antigen combinations, such as C-E-K- may be used to fill a request for say E-K-. However, amalgamating such antigen combinations would increase the probabilities, but not change the number of donors to be allocated to amalgamated antigen combinations. It is not expected that this will change the calculation of the number of donors needed to fulfil requests. In addition, some resolution information would be lost.

The analysis below will show that it is the large number of requests that are associated with antigen combinations with small probabilities that significantly increases the number of typed donors needed to fulfil the requests.

Figure 2: Probabilities for 403 genotype antigen combinations, with the horizontal axis showing antigen combination and the vertical axis showing probability

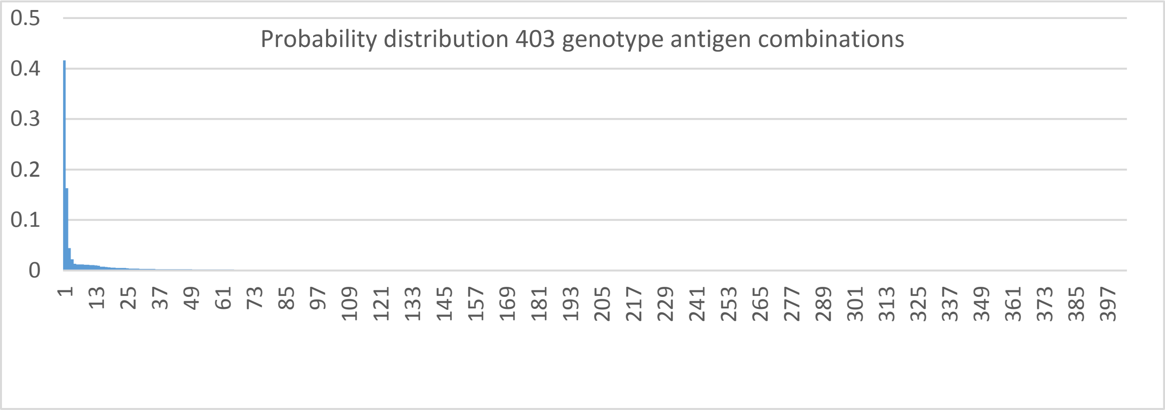

Figure 3: Probabilities for 403 genotype antigen combinations, with the horizontal axis showing antigen combination 26 to 403 and the vertical axis showing probability.

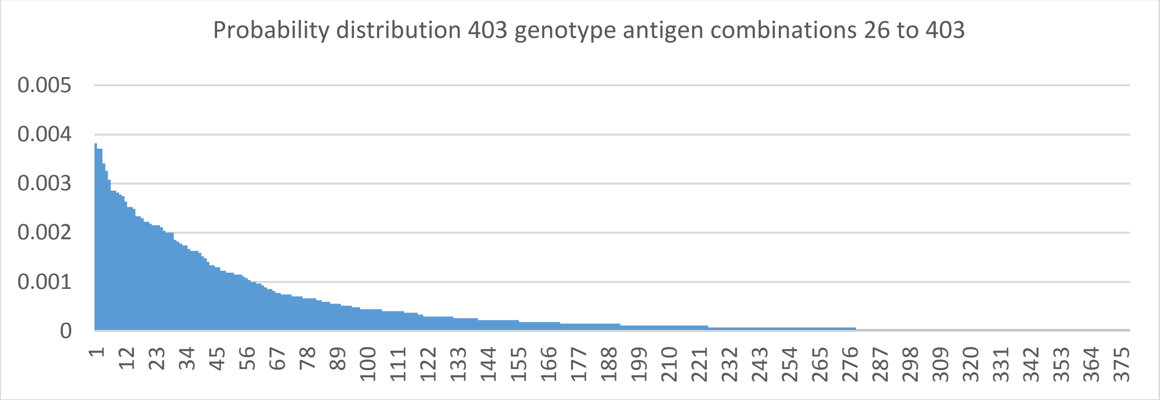

The bounds of Mallows in Theorem 2 were used to estimate that 205,000 donors need to be genotyped to obtain at least one donor for each of the 403 antigen combinations with 95% probability. This equates to 38% of the current donor panel. Since these lower and upper bounds differ by only 0.12% at the 95% confidence level, this indicates a high level of accuracy. A summary of the results is provided in Table 3.

With respect to phenotype testing, the number of distinct antigen combinations reduced to 299, with the probabilities recalibrated for the phenotype specific data. Using the same procedure, the number of donors to be phenotyped to obtain at least one donor for each of the 299 antigen combinations, with 95% probability, was estimated to be 35% of the donor panel or 188,000. A summary of the results is provided in Table 4. Once again there is a high degree of confidence in this estimate as the upper and lower bounds agree within 0.12%.

Table 3: Number of donors to be genotyped to achieve 95% and 85% confidence of obtaining at least one donor of each antigen combination. Estimates given to 3 significant figures.

Genotype estimates for donor panel of size 542,891 over 403 antigen combinations					
Confidence level, C	$S_{C:}$ Number of donors to achieve confidence	Percentage donor panel	Lower bound	Upper bound	Range of bounds
95%	205,000	37.76%	0.950001	0.951219	0.0012
85%	175,300	32.29%	0.850003	0.860614	0.0106

Table 4: Number of donors to be phenotyped to achieve 95% and 85% confidence of obtaining at least one donor of each antigen combination. Estimates given to 4 significant figures.

Phenotype estimates for donor panel of size 542,891 over 299 antigen combinations					
Confidence level, *C*	$S_{C:}$ Number of donors to achieve confidence	Percentage donor panel	Lower bound	Upper bound	Range of bounds
95%	188,000	34.60%	0.950007	0.951214	0.001207
85%	158,200	29.14%	0.850012	0.860536	0.0105

Validation of our bounds approach was conducted by performing Monte Carlo Multinomial simulations, as described previously. The results of this process are given in Figure 4 and

Figure 5. For Figure 4, we have assumed 23541 random donors are genotyped and three distinct Monte Carlo simulations scenarios have been run. Each group of four bars represents one antigen combination and each colour represents a single assignment of donors to antigen combinations. The target requests are indicated by light blue bars with the three Monte Carlo simulations shown in different colours. There are approximately 10000 requests for the first antigen combination (c-E-K-) and the sampling process has allocated most of the donors to that antigen combination, but not enough to meet all the targets. It can also be seen that the target for the antigen combination denoted 9 has not been met (as highlighted in the insert in Figure 4). This is true for many of the other antigen combinations, but some targets have been exceeded.

Figure 4: Request targets for a 120 day period shown in light blue, with three Monte Carlo Multinomial simulations where 23,541 donors (total number of requests) have been allocated to the 50 antigen combinations with the highest probabilities.

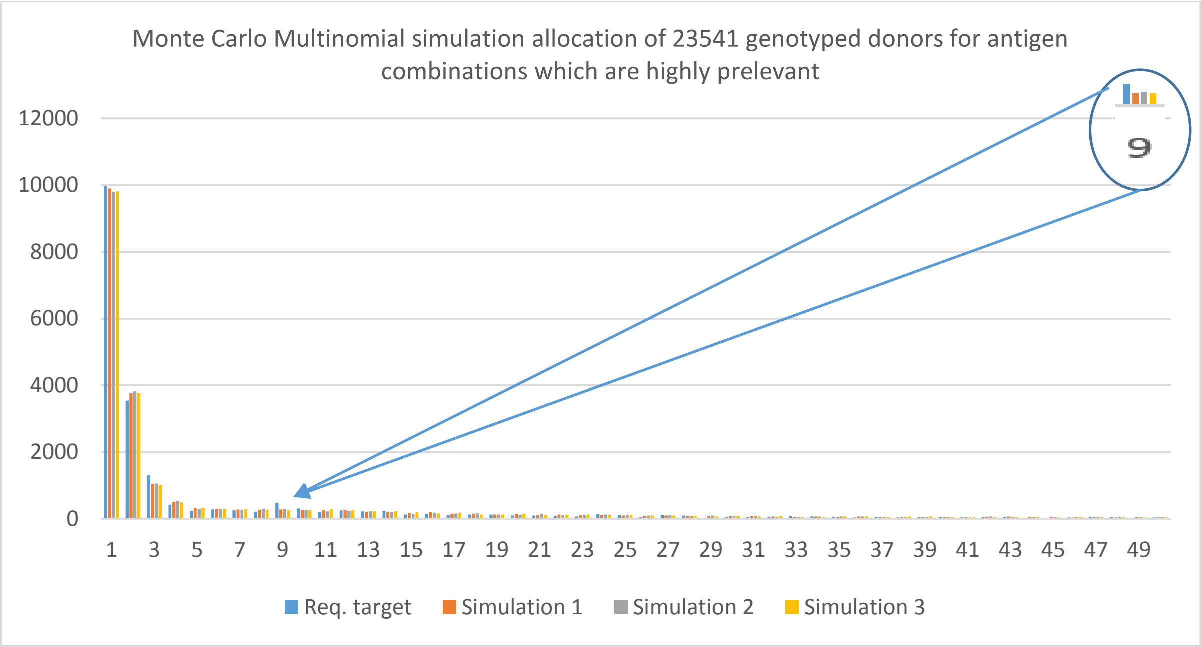
Monte Carlo Multinomial simulation allocation of 23541 genotyped donors for antigen combinations which are highly prelevant
12000
10000
8000
6000
4000
2000
0
1 3 5 7 9 11 13 15 17 19 21 23 25 27 29 31 33 35 37 39 41 43 45 47 49
9
Req. target
Simulation 1
Simulation 2
Simulation 3

Figure 5: Request targets for a 120 day period shown in light blue, with three Monte Carlo Multinomial simulations where 23,541 donors (total number of requests) have been allocated to the 50 antigen combinations with the least probabilities

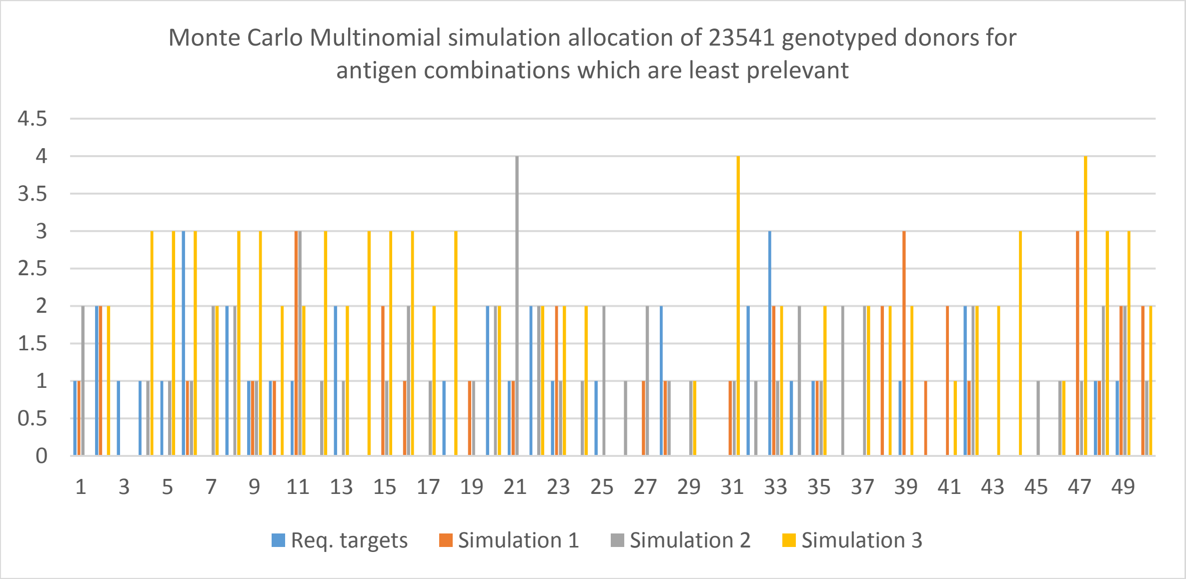

To meet a greater number of targets, the number of typed donors was increased to 47,082. Figure 6 and Figure 7 show the results of the simulations with 47,[illegible]2 typed donors.

Figure 6: Request targets for 120 days shown in light blue, with three Monte Carlo Multinomial simulations where 47,082 donors (twice the total number of requests) have been allocated to the 50 antigen combinations with highest probabilities

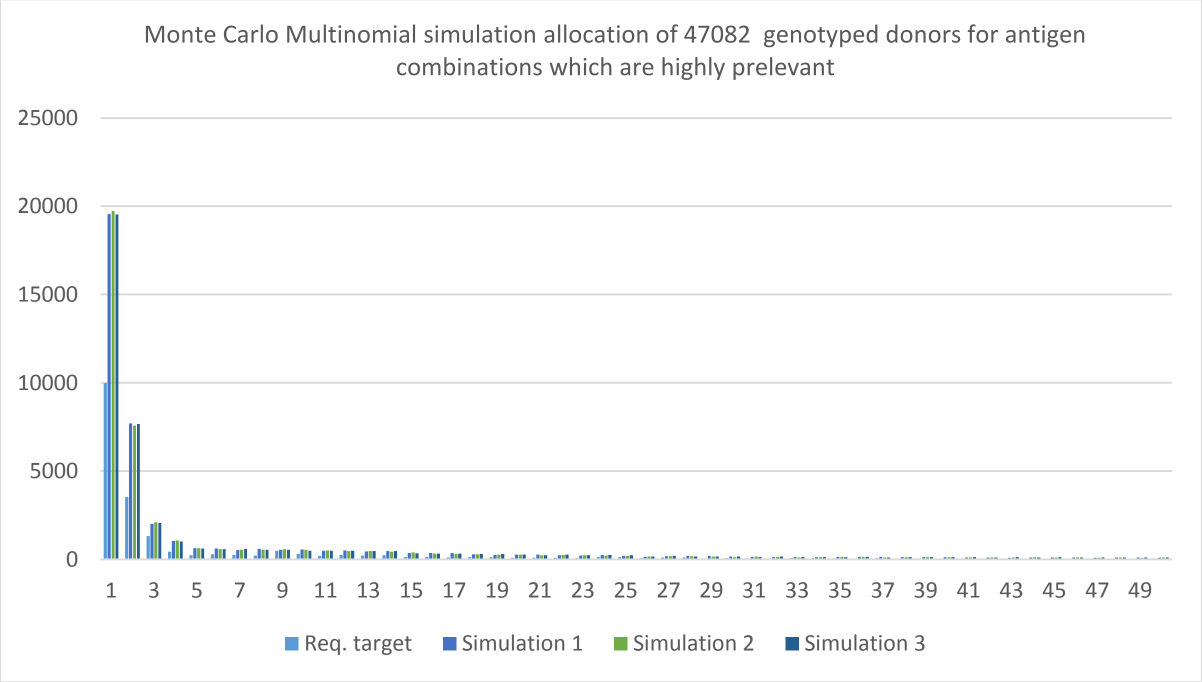

Figure 7: Request targets for 120 days shown in light blue, with three Monte Carlo Multinomial simulations where 47,082 donors (twice the total number of requests) have been allocated to the 50 antigen combinations with the least probabilities

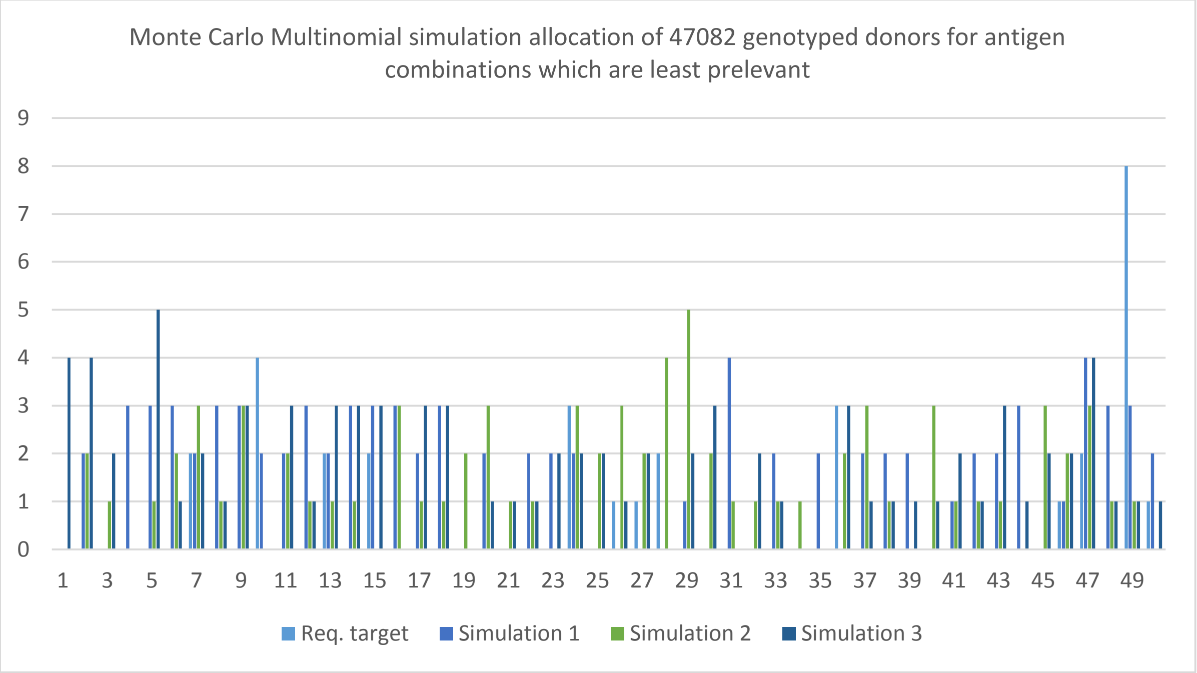

The attainment of targets with a confidence level of 95% is validated by demonstrating that the number of times the allocation of donors to antigen combinations fails to meet the requisite targets as a percentage of the number of antigen combinations over 10,000 Monte Carlo Multinomial simul[illegible]ons (data in Figure 4) m[illegible]ts the 95% confidence level.

To investigate the attainment of a 95% confidence interval further Monte Carlo Multinomial simulations were run. Targets for each of the 403 antigen combinations were determined for a given time period. Here these targets will be denoted $Kvec =$ $(k_1, \cdots, k_{403})$ with the sum of the requests denoted $K = k_1 + \cdots + k_{403}$. The specific [illegible]e periods that were chosen are:

I. 15 consecutive 8 day periods (a possible preferred period for holding blood in inventory), and
II. 3 consecutive 120 day periods (assuming donors donate up to 3 times per year).

As demonstrated in Figure 6 and Figure 7 if only *K* donors are genotyped then the probability of meeting the targets $(k_1, \cdots, k_{403})$ is close to 0. However, as the number of

genotyped donors increases, to *6*K*, the probability of meeting targets is in excess of 94%, as shown in Table 5.

Table 5: The probability of meeting targets based on fifteen 8 day periods and three 120 day periods, respectively over 10,000 Monte Carlo Multinomial simulations

Monte Carlo Multinomial simulation genotype			
Targets based on 8 day periods		Targets based on 120 day periods	
Number typed donors, S	Minimum success %	Number typed donors, S	Minimum success %
$6 * K$ =12900	[illegible].54%	$4 * K$ =94164	95.04%
$7 * K$ =15050	95.04%	$5 * K$ =117705	96.53%
$8 * K$ =17200	95.53%	$6 * K$ =141246	97.27%
$9 * K$ =19350	95.53%	$7 * K$ =164787	97.77%
$10 * K$ =21500	96.03%	$8 * K$ =188328	98.51%

The slight disparity between these numbers with the estimate of genotyping 205,000 donors (Table 3) is to be expected as in a 120 day period not all rare antigen combinations will be requested, however the Monte Carlo Multinomial simulation must still allocate donors to these antigen combinations just in case they are needed.

The estimates provided in Table 4 have been validated with 10,000 Monte Carlo Multinomial simulations where the phenotyping request targets have been determined for 8 day and 120 day periods. The results are given in Table 6 where the requests are represented by $Kvec = (k_1, \cdots, k_{299})$ and $K = k_1 + \cdots + k_{299}$. Once again results are consistent with the estimated number of donors to be phenotyped to achieve 95% confidence levels as in Table 4.

Table 6: The probability of meeting targets set from fifteen 8 day periods and three 120 day periods over 10,000 Monte Carlo Multinomial simulations

Monte Carlo Multinomial simulation phenotype			
Targets set over 8 day periods		Targets set over 120 day periods	
Number of type donors typed, S	Minimum success Percentage	Number of typed donors typed, S	Minimum success Percentage
$4 * K$ =12888	93.98%	$4 * K$ =94156	95.32%
$5 * K$ =15036	94.65%	$5 * K$ =117695	95.99%
$6 * K$ =17184	94.98%	$6 * K$ =141234	97.32%

$7 * K$ =19332	95.32%	$7 * K$ =164773	97.66%
$8 * K$ =21480	95.65%	$8 * K$ =188312	98.66%

For the purpose of illustrating the techniques presented here, the calculation of timelines for meeting genotyping levels are based on the following assumptions.

- Weekly donations are collected from 13,500 returning donors and 1% of these are genotyped per week.
- Currently $q = 13500 * 0.01 * 365/7 = 7{,}039$ donors genotyped each year.
- The current number of donors genotyped is $D_0 = 0.018 * 542891 = 9772$.
- *y* represents the number of years of genotyping.
- *m* represents the percentage of non-returning donors per annum, m=0.13, i.e. 13%.

All calculations are again based on the formula

$$D_y = (1-m)^y D_0 + \frac{M}{m}(1-(1-m)^y),$$

Error! Reference source not found. Table 7 shows the effect on the total number of donors genotyped for various typing percentages. Due to the assumed annual loss factor of 13% for non-returning donors, annual genotyping levels of 1% or even 4% are not enough to attain a cumulative 38% level for genotyping the donor panel within 20 years. However, if the level of genotyping is raised to 5% then 38% or 205,000 genotyped donors would be reached within 12 years, and if 12% of the donor panel is genotyped each year then 38% will be attained within 3 years.

Furthermore, once a level of 38% genotyping of the donor panel is reached and given that 13% of donors do not return, then an annual genotyping level of 2.14% of the donor panel will maintain this level at 38%.

Table 7 Estimates (rounded to integers) of the number of years needed to reach 205,000 genotyped donors at the given percentages, minimum values that exceed target are shown

Given percentage of genotyping, the number of donors that will be genotyped after *y* years									
	Genotyping percentage of donor panel								
	≤ 4%	5%	6%	7%	8%	9%	10%	11%	12%
Years to attainment	Never attained	12	8	7	5	5	4	4	3
Number typed donors	N/A	207381	207277	224388	208024	233419	221834	243458	213909

The calculation of timelines for meeting phenotyping levels are based on the assumptions.

- Weekly donations are collected from 13,500 returning donors and 5% of these are phenotyped each week.
- Currently $q = 13500 * 0.05 * 365/7 = 35196$ donors are phenotyped each year.
- The current number of donors phenotyped is $D_0 = 542{,}891 * 0.121 = 65689$.
- *y* represents the number of years of phenotyping.
- *m* represents the percentage of non-returning donors per annum, *m=0.13,* 13%.

Table 3 gives estimates of the effect of increasing the percentage of phenotyping. If the level of phenotyping is retained at 5% of the donor panel, then 35% of the donor panel (188,000 donors) will be phenotyped within 8 years and if the level of phenotyping is increased to 9% of the donor panel, then a 35% target will be reached in 3 years. Furthermore, once a level of 35% phenotyping of the donor panel is reached and given that 13% of donors are not returning, then an annual phenotyping level of 1.94% of the donor panel will maintain this level at 35%.

Table 3: Estimates (rounded to integers) of the number of years needed to reach 188,000 phenotyped donors at the given percentages, minimum values that exceed target are shown

Given percentage of phenotyping, the number of donors that will be phenotyped after y years						
	Phenotyping percentage of donor panel					
	≤ 4%	5%	6%	7%	8%	9%
Years to attainment	Never attained	8	6	4	4	3
Number typed donors	N/A	191619	200533	188999	210622	198862

In conclusion we have used a Multinomial distribution to provide estimates for how many donors should be tested to meet Lifeblood's requests with respect to genotyping and phenotyping.

Genotyping of 38% of the donor panel is required to meet demand with at least a 95% confidence level. At the current level of 1% genotyping of weekly inventory, it is estimated that it is not possible to reach a target of 38% (205,000 donors). If the weekly genotyping levels are raised to 5% the target will be reached within 12 years. Increasing to 12% would allow the 38% target to be reached within 3 years. The model also demonstrated that when the target is reached then annual genotyping of an additional 28,503 is required to maintain the overall level.

Phenotyping of 35% of the donor panel is required to meet Lifeblood's demand with at least a 95% confidence level. At the current level of 5% phenotyping of inventory, it is estimated that it will take 8 years to reach a target of 35% (188,000). If the level of extended phenotyping of weekly returning donors is raised to 9% then a target would be achieved within 3 years. The model also demonstrated that when the target is reached then annual extended phenotyping of an additional 26,140 is required to maintain the overall level.

Conclusion

The mathematical model was constructed based on recent historical blood request data for uncommon red cell antigen combinations, supplied by Lifeblood. A probabilistic model based on Multinomial distribution was developed and validated by our team to determine the testing regimes required to meet targets within certain timeframes.

According to this model, the current demand for phenotype matched red cells would be met within three years if 9% of weekly returning donors were phenotyped, and maintained if over 26,000 additional donors were phenotyped each subsequent year. Genotyping 12% of weekly returning donors would reach the target within 3 years and be maintained if over 28,500 additional donors were genotyped each subsequent year. This mathematical model will inform business decisions and assist Lifeblood to determine the level of investment required to meet the desired timeframe to achieve the optimum panel size.

Acknowledgement:

Australian governments fund Australian Red Cross Lifeblood for the provision of blood, blood products and services to the Australian community.

References

[1] Lomas-Francis C. Testing for rare blood donors by DNA analysis. ISBT Science Series. 2006;1:213-219.

[2] Flegel W, Gottschall J, Denomme G. Implementing mass-scale red cell genotyping at a blood center. Transfusion. 2015;55(11):2610-2615.

[3] Sapatnekar S. Molecular Typing for Red Blood Cell Antigens. The American Association for Clinical Chemistry. 2015 Mar.

[4] Hustinx H. DGTI register of rare donors. Transfus Med Hemother. 2014;41(5):338-341.

[5] Portegysa J, Rinka G, Bloosa P, Scharbergb EA, Klutera H, Bugerta P. Towards a Regional Registry of Extended Typed Blood Donors: Molecular Typing for Blood Group, Platelet and Granulocyte Antigens. Transfus Med Hemother 2018;45:331-340 DOI: 10.1159/000493555.

[6] Blake JT, Clarke G. Modeling rare blood in Canada. Transfusion. 2019;59:582-592.

[7] Perreault J, Lavoie J. Painchaud P, Cote M, Constanzo-Yanez J, Cote R et al. Set-up and routine use of a database of 10 555 genotyped blood donors to facilitate the screening of compatible blood components for alloimmunized patients. Vox Sanguinis. 2009;97:61-68.

[8] Denomme G, Anani W, Avent N, Bein G, Briggs L, Lapadat R et al. Red cell genotyping precision medicine: a conference summary. Therapeutic Advances in Hematology. 2017;8(10):227-291.

[9] Reesink H, Engelfriet C, Schennach H, Gassner C, Wendel S, Fonao-Wendel R et al. Donors with a rare pheno (geno) type. Vox Sanguinis. 2008;95:236-253..

[10] International Society of Blood Transfusion Working Party on Rare Donors. Immunohematology. Part 1: International Rare Donor Programs. J Blood Group Sero and Mol Genetics. 2016;32(1).

[11] Mallows CL. An inequality involving multinomial probabilities. Biometrika. 1968:422-424.

Supplement

Antigen Combination Analysis

Table 4: Antigen combinations that are met only by extended phenotyping

a) Antigen combination data specific to extended phenotyping							
Antigen combination	Total num. req. units	Total num. of req.	Fraction of 27001	Antigen combination	Total num. req. units	Total num. of req.	Fraction of 27001
c-E-K-	30673	11280	0.41776	C-E-Fya-K-s-	11	6	0.00022
C-E-K-	10346	4409	0.16329	C-E-Fyb-K-S-	13	6	0.00022
E-K-	3711	1235	0.04574	c-Fya-K-	23	6	0.00022
C-e-K-	1459	590	0.02185	C-Fyb-Jkb-K-	8	6	0.00022
K-M-	707	352	0.01304	C-Jkb-K-S-	13	6	0.00022
E-Fya-K-	1004	338	0.01252	E-Fyb-Jkb-K-s-	6	6	0.00022
Fya-K-	810	334	0.01237	E-M-	12	6	0.00022
C-E-Fya-Jkb-K-	604	318	0.01178	Fya-K-M-S-	10	6	0.00022
K-S-	1041	308	0.01141	Fyb-Jkb-K-	11	6	0.00022
Jka-K-	673	301	0.01115	Jka-K-S-	13	6	0.00022
c-E-Jka-K-	748	297	0.011	Jkb-S-	15	6	0.00022
C-K-	659	294	0.01089	M-S-	14	6	0.00022
c-E-Fya-K-	689	282	0.01044	C-e-k-	8	5	0.00019
E-Jka-K-	653	270	0.01	c-E-M-	10	5	0.00019
Jka-	461	206	0.00763	C-Fya-Jkb-K-	5	5	0.00019
K-	554	204	0.00756	c-Jka-K-	13	5	0.00019
Fya-	499	196	0.00726	C-K-s-	11	5	0.00019
c-E-Jkb-K-	383	184	0.00681	E-Fyb-	16	5	0.00019
C-E-Fya-K-	430	183	0.00678	E-Fyb-Jkb-K-M-S-	6	5	0.00019
c-E-	435	160	0.00593	C-E-Fya-K-M-S-	8	4	0.00015
E-Jkb-K-	419	159	0.00589	C-E-Fyb-Jka-K-S-	9	4	0.00015
M-	367	156	0.00578	C-Fya-Jka-K-S-	7	4	0.00015
C-E-Jka-K-	329	142	0.00526	C-Fya-Jkb-S-	9	4	0.00015
C-Fya-K-	319	142	0.00526	C-S-	13	4	0.00015
c-E-Fyb-Jka-K-	299	132	0.00489	E-Fya-Jka-S-	5	4	0.00015
C-e-	340	119	0.00441	E-Fya-Jkb-	9	4	0.00015
Jkb-K-	274	110	0.00407	e-Jka-K-	10	4	0.00015
c-E-Fyb-Jkb-K-	219	108	0.004	E-Jka-K-M-S-	10	4	0.00015
c-E-Fyb-K-	214	104	0.00385	E-Jkb-K-M-	6	4	0.00015
C-E-Jkb-K-	215	96	0.00356	Fya-Jka-K-M-S-	6	4	0.00015
E-Fya-Jkb-K-	276	96	0.00356	Fya-Jkb-K-S-	8	4	0.00015

E-Fyb-Jkb-K-	203	92	0.00341	Fya-M-S-	7	4	0.00015
E-	222	87	0.00322	Jka-M-	4	4	0.00015
E-K-S-	215	80	0.00296	C-E-Fya-Jka-K-S-	4	3	0.00011
c-E-Fya-Jkb-K-	200	79	0.00293	C-E-Fya-K-M-	7	3	0.00011
C-Jka-K-	187	79	0.00293	c-E-Fyb-	7	3	0.00011
c-K-	191	77	0.00285	c-E-Fyb-K-M-S-	4	3	0.00011
E-Fyb-K-	196	76	0.00281	c-E-Jka-K-M-	4	3	0.00011
C-Jkb-K-	191	74	0.00274	C-e-Jka-K-S-	6	3	0.00011
E-Fya-Jka-K-	201	69	0.00256	C-e-Jkb-K-S-	5	3	0.00011
C-e-Jka-K-	143	68	0.00252	C-E-S-	7	3	0.00011
E-K-M-	159	68	0.00252	C-Fya-Jkb-	5	3	0.00011
Fya-Jkb-K-	160	63	0.00233	c-Fyb-K-	6	3	0.00011
E-Jka-K-S-	130	62	0.0023	c-Fyb-K-S-	9	3	0.00011
C-E-	149	60	0.00222	C-Jka-K-M-S-	6	3	0.00011
C-E-Fya-K-S-	159	60	0.00222	C-Jka-K-S-	6	3	0.00011
Fyb-Jka-K-	114	60	0.00222	c-K-M-	4	3	0.00011
C-E-K-S-	116	59	0.00219	E-Fya-Jka-K-M-S-	6	3	0.00011
C-E-Fya-Jkb-K-S-	155	58	0.00215	E-Fya-Jka-K-s-	5	3	0.00011
c-E-Fya-K-S-	119	55	0.00204	E-Fya-k-	4	3	0.00011
E-Fya-K-S-	126	55	0.00204	E-Fya-s-	6	3	0.00011
E-Jka-	125	55	0.00204	E-Fyb-Jka-K-s-	5	3	0.00011
E-Jkb-K-S-	112	55	0.00204	E-Jka-s-	3	3	0.00011
c-E-K-M-	118	54	0.002	Fya-M-	10	3	0.00011
Fyb-K-	131	54	0.002	Jka-Jkb-	4	3	0.00011
c-E-K-S-	116	51	0.00189	Jka-Jkb-K-	4	3	0.00011
C-	152	49	0.00181	Jka-K-M-	3	3	0.00011
C-e-Fyb-K-	84	49	0.00181	c-E-Fya-Jka-	2	2	0.00007
c-E-Jkb-K-S-	99	48	0.00178	C-e-Fya-Jkb-	3	2	0.00007
C-E-Fyb-K-	87	46	0.0017	c-E-Fya-K-M-	5	2	0.00007
C-E-Fya-Jka-K-	130	44	0.00163	c-E-Fya-M-S-	2	2	0.00007
E-Fya-Jkb-K-S-	89	44	0.00163	C-E-Fya-S-	6	2	0.00007
C-e-Fya-K-	92	43	0.00159	C-e-Fyb-Jka-K-	3	2	0.00007
E-Fya-	100	40	0.00148	c-E-Fyb-K-M-	9	2	0.00007
C-Fya-K-S-	94	39	0.00144	c-E-Jka-K-M-S-	2	2	0.00007
C-K-S-	92	39	0.00144	c-E-Jka-K-s-	6	2	0.00007
C-E-Jkb-K-S-	64	38	0.00141	C-E-Jkb-S-	4	2	0.00007
c-E-Jka-K-S-	72	37	0.00137	C-e-K-M-S-	12	2	0.00007
e-	118	36	0.00133	C-e-K-s-	6	2	0.00007
c-E-Fyb-K-S-	69	35	0.0013	c-E-s-	4	2	0.00007
c-E-Fya-Jka-K-	79	34	0.00126	c-Fya-	4	2	0.00007
c-E-Fya-Jkb-K-S-	60	33	0.00122	C-Fya-Jka-S-	3	2	0.00007
C-E-K-M-	73	33	0.00122	C-Fya-S-	6	2	0.00007

c-E-Jka-	66	32	0.00119	c-Fyb-Jka-K-	2	2	0.00007
E-Fya-K-s-	49	32	0.00119	C-Fyb-Jka-K-M-S-	4	2	0.00007
E-Fyb-Jkb-K-S-	49	31	0.00115	C-Fyb-Jkb-K-S-	4	2	0.00007
E-K-s-	69	31	0.00115	C-Fyb-K-S-	4	2	0.00007
Jkb-	86	28	0.00104	c-Jka-	5	2	0.00007
C-E-Fyb-Jkb-K-	67	27	0.001	C-Jka-K-s-	3	2	0.00007
C-e-Jka-	57	27	0.001	c-Jkb-K-	4	2	0.00007
E-Fya-Jka-K-S-	46	26	0.00096	C-k-	2	2	0.00007
E-Fyb-Jka-K-S-	49	26	0.00096	C-K-M-S-	8	2	0.00007
E-Fyb-Jka-K-	42	25	0.00093	C-M-	3	2	0.00007
E-Jkb-	58	25	0.00093	E-Fyb-K-M-	6	2	0.00007
C-e-Jkb-K-	42	23	0.00085	E-Jkb-s-	2	2	0.00007
Fya-Jka-K-	50	23	0.00085	e-K-M-	4	2	0.00007
S-	58	23	0.00085	E-M-S-	5	2	0.00007
E-Fya-K-M-S-	39	22	0.00081	Fya-Jka-K-S-	4	2	0.00007
c-E-Fya-	53	21	0.00078	Fyb-Jkb-K-s-	4	2	0.00007
Fyb-Jka-	38	21	0.00078	Fyb-Jkb-K-S-	5	2	0.00007
C-Fyb-Jka-K-	32	20	0.00074	Fyb-K-M-	3	2	0.00007
C-e-Fya-	59	19	0.0007	Jka-s-	2	2	0.00007
C-Fya-Jka-K-	26	19	0.0007	Jkb-k-	2	2	0.00007
E-Fyb-K-S-	40	19	0.0007	Jkb-K-s-	3	2	0.00007
Fya-Jka-	54	19	0.0007	Jkb-s-	2	2	0.00007
K-s-	34	19	0.0007	s-	6	2	0.00007
C-e-K-M-	29	18	0.00067	C-E-Fya-Fyb-Jka-K-	4	1	0.00004
C-Jkb-K-s-	33	18	0.00067	C-E-Fya-Jka-K-M-	1	1	0.00004
C-E-Jkb-	43	17	0.00063	C-E-Fya-Jka-K-s-	1	1	0.00004
C-K-M-	27	17	0.00063	C-E-Fya-Jkb-	1	1	0.00004
C-E-Fyb-Jkb-K-S-	28	16	0.00059	c-E-Fya-Jkb-K-M-S-	4	1	0.00004
c-E-K-M-S-	31	16	0.00059	c-E-Fya-Jkb-S-	4	1	0.00004
Jkb-K-S-	29	16	0.00059	c-E-Fya-s-	4	1	0.00004
C-E-Fyb-Jka-K-	24	15	0.00056	C-E-Fya-s-	1	1	0.00004
C-Jka-	37	15	0.00056	C-E-Fyb-Jka-K-s-	1	1	0.00004
Fyb-	35	15	0.00056	c-E-Fyb-Jka-S-	2	1	0.00004
c-E-Fya-Jka-K-S-	23	14	0.00052	C-E-Fyb-Jka-S-	2	1	0.00004
c-E-Fyb-Jkb-K-S-	24	14	0.00052	c-E-Fyb-Jkb-	3	1	0.00004
e-K-	35	14	0.00052	C-e-Fyb-K-S-	1	1	0.00004
E-k-	17	14	0.00052	c-E-Jka-Jkb-K-	1	1	0.00004
K-M-S-	38	14	0.00052	C-E-Jka-K-M-S-	3	1	0.00004
c-E-Fyb-Jka-K-S-	18	13	0.00048	C-e-Jka-K-s-	1	1	0.00004
c-E-S-	40	13	0.00048	c-E-Jka-S-	2	1	0.00004
C-E-Fya-	31	12	0.00044	C-E-Jka-S-	1	1	0.00004
C-E-Jka-K-S-	25	12	0.00044	c-E-Jkb-S-	2	1	0.00004

C-e-K-S-	25	12	0.00044
C-Fyb-K-	35	12	0.00044
E-Fya-K-M-	23	12	0.00044
E-S-	31	12	0.00044
Fya-Jkb-K-s-	21	12	0.00044
Fyb-K-S-	31	12	0.00044
c-E-Jkb-	27	11	0.00041
c-Jkb-	23	11	0.00041
E-Jkb-K-s-	18	11	0.00041
E-K-M-S-	23	11	0.00041
k-	18	11	0.00041
Fya-K-S-	23	10	0.00037
c-E-Fya-Jkb-	13	8	0.0003
c-E-Fya-K-s-	16	8	0.0003
C-E-Jka-	15	8	0.0003
C-E-Jkb-K-M-	22	8	0.0003
C-e-S-	14	8	0.0003
C-Fya-	18	8	0.0003
C-Fya-K-s-	13	8	0.0003
E-Fya-S-	24	8	0.0003
E-Fyb-Jka-	12	8	0.0003
E-Jka-K-M-	16	8	0.0003
Fya-Jkb-	17	8	0.0003
Jka-S-	19	8	0.0003
C-E-Fya-Jka-K-M-S-	7	7	0.00026
C-e-Fya-Jkb-K-	18	7	0.00026
c-E-Fya-Jkb-K-s-	8	7	0.00026
c-E-Fya-K-M-S-	11	7	0.00026
C-e-Fya-K-S-	13	7	0.00026
c-E-Fya-S-	14	7	0.00026
C-E-Jkb-K-s-	13	7	0.00026
C-E-K-M-S-	17	7	0.00026
E-Fyb-K-s-	12	7	0.00026
Fya-S-	17	7	0.00026
c-	26	6	0.00022
C-E-K-Jka-	3	1	0.00004
c-E-k-S-	2	1	0.00004
C-E-K-s-	2	1	0.00004
C-e-M-	2	1	0.00004
C-E-M-	2	1	0.00004
C-E-M-S-	2	1	0.00004
C-Fya-Jka-M-S-	1	1	0.00004
c-Fya-Jkb-K-	2	1	0.00004
C-Fya-Jkb-K-S-	3	1	0.00004
c-Fya-K-S-	2	1	0.00004
C-Fyb-	2	1	0.00004
C-Fyb-Jka-K-S-	1	1	0.00004
c-Jka-M-	2	1	0.00004
C-Jka-M-	2	1	0.00004
C-Jkb-	4	1	0.00004
c-Jkb-K-S-	4	1	0.00004
c-K-S-	2	1	0.00004
c-S-	3	1	0.00004
C-s-	2	1	0.00004
E-Fyb-Jkb-S-	1	1	0.00004
E-Fyb-K-M-S-	3	1	0.00004
E-Jka-K-s-	1	1	0.00004
E-Jka-M-	2	1	0.00004
E-Jkb-K-M-S-	2	1	0.00004
E-Jkb-S-	2	1	0.00004
e-K-M-S-	1	1	0.00004
Fya-Jka-K-M-	1	1	0.00004
Fya-Jkb-K-M-S-	1	1	0.00004
Fya-K-s-	1	1	0.00004
Fyb-Jka-K-s-	3	1	0.00004
Fyb-Jka-K-S-	4	1	0.00004
Fyb-Jkb-K-M-	1	1	0.00004
Fyb-S-	2	1	0.00004
Jka-K-M-S-	2	1	0.00004

Table 5: Antigen combination that are met only by genotyping

b) Antigen combinations that are able to be met **only** by genotyping

Antigen combination	Total num. of req. units	Total num. of req.	Fraction of 27001	Antigen combination	Total num. of req. units	Total num. of req.	Fraction of 27001
K-Kpa-	275	100	0.0037	E-Fya-K-Lua-	5	2	0.00007
c-E-K-Kpa-	163	47	0.00174	E-Fyb-Jka-K-Kpa-s-	4	2	0.00007
K-Lua-	142	41	0.00152	E-K-Lua-N-	3	2	0.00007
E-K-Kpa-	91	29	0.00107	Fya-Jka-K-Kpa-	2	2	0.00007
E-Fya-Jkb-K-Lua-	90	21	0.00078	Fya-K-Lua-	6	2	0.00007
c-E-Fya-K-Kpa-	55	20	0.00074	Fya-K-N-	3	2	0.00007
E-Jkb-K-Kpa-	39	20	0.00074	Kpa-	4	2	0.00007
E-K-Kpa-S-	56	18	0.00067	C-Cob-E-Jka-K-Kpa-Lua-S-	1	1	0.00004
c-E-Fyb-K-Kpa-	45	16	0.00059	C-Cob-Fya-K-S-	2	1	0.00004
C-E-Jkb-K-Kpa-	27	13	0.00048	C-Cob-K-Kpa-	2	1	0.00004
C-e-Fya-K-Kpa-	34	12	0.00044	c-Doa-E-Jka-K-Kpa-S-	1	1	0.00004
C-E-Jkb-K-Lua-	31	12	0.00044	C-E-Fya-Jkb-K-N-	1	1	0.00004
Fya-K-Kpa-	21	11	0.00041	c-E-Fya-K-Kpa-M-S-	1	1	0.00004
C-E-K-Kpa-	23	10	0.00037	C-E-Fya-K-Kpa-N-	1	1	0.00004
c-E-K-Kpa-M-	20	10	0.00037	c-E-Fya-K-Kpa-S-	3	1	0.00004
E-Jkb-K-Kpa-S-	14	10	0.00037	C-E-Fya-K-N-s-	2	1	0.00004
Jkb-K-Lua-	21	10	0.00037	c-E-Fya-Lub-	1	1	0.00004
c-E-K-Lua-	22	9	0.00033	C-E-Fya-N-s-	1	1	0.00004
E-K-Lua-	21	9	0.00033	C-E-Fyb-K-Kpa-	2	1	0.00004
C-E-Jkb-K-N-	17	8	0.0003	c-E-Jka-K-Kpa-	6	1	0.00004
N-	13	7	0.00026	C-E-Jka-K-N-	2	1	0.00004
E-K-N-	16	6	0.00022	C-E-Jka-Kpa-S-	1	1	0.00004
C-E-Fya-K-Kpa-	11	5	0.00019	c-E-Jkb-K-Kpa-	6	1	0.00004
c-E-Fyb-Jkb-K-Kpa-	26	5	0.00019	C-E-Jkb-K-Kpa-S-	1	1	0.00004
C-E-Jka-K-Kpa-S-	11	5	0.00019	C-E-Jkb-K-N-S-	3	1	0.00004
C-Jka-K-Lua-	22	5	0.00019	C-E-K-Kpa-Lua-	3	1	0.00004
E-Fya-K-Kpa-	17	5	0.00019	C-E-K-Lua-	2	1	0.00004
K-N-	12	5	0.00019	C-E-K-Lua-M-	2	1	0.00004
C-Cob-E-K-	14	4	0.00015	c-E-K-Lua-S-	2	1	0.00004
c-E-Fya-Jka-K-Kpa-	23	4	0.00015	c-E-K-N-	2	1	0.00004
c-E-Fya-K-Lua-	24	4	0.00015	C-e-K-N-	1	1	0.00004
C-E-Jkb-K-Kpa-M-	10	4	0.00015	C-Fya-N-S-	2	1	0.00004
c-E-K-Lub-	8	4	0.00015	C-Jkb-K-N-S-	1	1	0.00004

C-Fya-K-N-s-	7	4	0.00015	C-K-Lua-	2	1	0.00004
C-K-N-S-	12	4	0.00015	C-K-N-	2	1	0.00004
E-Jka-K-N-S-	4	4	0.00015	C-Kpa-S-	4	1	0.00004
c-E-Jka-K-Kpa-S-	3	3	0.00011	C-Lua-	2	1	0.00004
C-E-K-N-	5	3	0.00011	C-N-	1	1	0.00004
c-E-Kpa-	7	3	0.00011	Cob-E-Fya-K-S-	2	1	0.00004
C-E-Kpa-	9	3	0.00011	Cob-E-Jka-K-Kpa-	2	1	0.00004
C-Jkb-K-Kpa-	9	3	0.00011	E-Fyb-Jka-K-Kpa-	2	1	0.00004
E-Lua-	4	3	0.00011	E-Fyb-K-Kpa-s-	3	1	0.00004
Fya-Jka-K-N-	4	3	0.00011	E-Fyb-K-N-S-	1	1	0.00004
Lua-	4	3	0.00011	E-Jkb-K-Lua-S-	5	1	0.00004
c-E-Fya-Jkb-K-Kpa-	12	2	0.00007	E-Jkb-K-N-S-	2	1	0.00004
C-E-Fya-K-Lua-	4	2	0.00007	E-Kpa-	2	1	0.00004
c-E-Jka-K-Lua-	16	2	0.00007	E-N-S-	4	1	0.00004
C-E-Jka-K-Lua-	3	2	0.00007	Fya-Jka-K-Kpa-S-	3	1	0.00004
c-E-Kpa-S-	6	2	0.00007	Jka-K-Kpa-	2	1	0.00004
C-Fya-Jka-K-N-S-	3	2	0.00007	Jka-K-Lua-	1	1	0.00004
C-Fya-K-Kpa-	5	2	0.00007	Jkb-K-Lua-S-	2	1	0.00004
C-Fya-K-N-	3	2	0.00007	K-Kpa-M-S-	8	1	0.00004
E-Fya-Jka-K-Lua-	2	2	0.00007	Kpb-	1	1	0.00004
					SUM	603	

Table 6: Determination of the frequency of the 106 antigen combinations that contain a genotyping specific antigen within the relevant class of extended phenotype combinations.

Analysis of frequency of genotype combinations within phenotype combinations					
Antigen combination	Extended phenotype antigen	Frequency of request	Genotype antigen	Frequency of request	Probability
C-Lua-	C-	49	Lua-	1	0.02041
C-N-	C-	49	N-	1	0.02041
c-E-Kpa-	c-E-	160	Kpa-	3	0.01875
C-E-Kpa-	C-E-	60	Kpa-	3	0.05
c-E-Fya-Lub-	c-E-Fya-	21	Lub-	1	0.04762
c-E-Fya-Jka-K-Kpa-	c-E-Fya-Jka-K-	34	Kpa-	4	0.11765
c-E-Fya-Jkb-K-Kpa-	c-E-Fya-Jkb-K-	79	Kpa-	2	0.02532
C-E-Fya-Jkb-K-N-	C-E-Fya-Jkb-K-	318	N-	1	0.00314
c-E-Fya-K-Kpa-	c-E-Fya-K-	282	Kpa-	20	0.07092
C-e-Fya-K-Kpa-	C-e-Fya-K-	43	Kpa-	12	0.27907
C-E-Fya-K-Kpa-	C-E-Fya-K-	183	Kpa-	5	0.02732
C-E-Fya-K-Kpa-N-	C-E-Fya-K-	183	Kpa-N-	1	0.00546
c-E-Fya-K-Lua-	c-E-Fya-K-	282	Lua-	4	0.01418
C-E-Fya-K-Lua-	C-E-Fya-K-	183	Lua-	2	0.01093
c-E-Fya-K-Kpa-M-S-	c-E-Fya-K-M-S-	7	Kpa-	1	0.14286
c-E-Fya-K-Kpa-S-	c-E-Fya-K-S-	55	Kpa-	1	0.01818
C-E-Fya-K-N-s-	C-E-Fya-K-s-	6	N-	1	0.16667
C-E-Fya-N-s-	C-E-Fya-s-	1	N-	1	1
c-E-Fyb-Jkb-K-Kpa-	c-E-Fyb-Jkb-K-	108	Kpa-	5	0.0463
c-E-Fyb-K-Kpa-	c-E-Fyb-K-	104	Kpa-	16	0.15385
C-E-Fyb-K-Kpa-	C-E-Fyb-K-	46	Kpa-	1	0.02174
c-E-Jka-K-Kpa-	c-E-Jka-K-	297	Kpa-	1	0.00337
c-E-Jka-K-Lua-	c-E-Jka-K-	297	Lua-	2	0.00673
C-E-Jka-K-Lua-	C-E-Jka-K-	142	Lua-	2	0.01408
C-E-Jka-K-N-	C-E-Jka-K-	142	N-	1	0.00704
C-Cob-E-Jka-K-Kpa-Lua-S-	C-E-Jka-K-S-	12	Cob-Kpa-Lua-	1	0.08333
c-Doa-E-Jka-K-Kpa-S-	c-E-Jka-K-S-	37	Doa-Kpa-	1	0.02703
c-E-Jka-K-Kpa-S-	c-E-Jka-K-S-	37	Kpa-	3	0.08108
C-E-Jka-K-Kpa-S-	C-E-Jka-K-S-	12	Kpa-	5	0.41667
C-E-Jka-Kpa-S-	C-E-Jka-S-	1	Kpa-	1	1
c-E-Jkb-K-Kpa-	c-E-Jkb-K-	184	Kpa-	1	0.00543
C-E-Jkb-K-Kpa-	C-E-Jkb-K-	96	Kpa-	13	0.13542
C-E-Jkb-K-Lua-	C-E-Jkb-K-	96	Lua-	12	0.125
C-E-Jkb-K-N-	C-E-Jkb-K-	96	N-	8	0.08333
C-E-Jkb-K-Kpa-M-	C-E-Jkb-K-M-	8	Kpa-	4	0.5
C-E-Jkb-K-Kpa-S-	C-E-Jkb-K-S-	38	Kpa-	1	0.02632

C-E-Jkb-K-N-S-	C-E-Jkb-K-S-	38	N-	1	0.02632
C-Cob-E-K-	C-E-K-	4409	Cob-	4	0.00091
c-E-K-Kpa-	c-E-K-	11280	Kpa-	47	0.00417
C-E-K-Kpa-	C-E-K-	4409	Kpa-	10	0.00227
C-E-K-Kpa-Lua-	C-E-K-	4409	Kpa-Lua-	1	0.00023
c-E-K-Lua-	c-E-K-	11280	Lua-	9	0.0008
C-E-K-Lua-	C-E-K-	4409	Lua-	1	0.00023
c-E-K-Lub-	c-E-K-	11280	Lub-	4	0.00035
c-E-K-N-	c-E-K-	11280	N-	1	0.00009
C-e-K-N-	C-e-K-	590	N-	1	0.00169
C-E-K-N-	C-E-K-	4409	N-	3	0.00068
c-E-K-Kpa-M-	c-E-K-M-	54	Kpa-	10	0.18519
C-E-K-Lua-M-	C-E-K-M-	33	Lua-	1	0.0303
c-E-K-Lua-S-	c-E-K-S-	51	Lua-	1	0.01961
c-E-Kpa-S-	c-E-S-	13	Kpa-	2	0.15385
C-Fya-Jka-K-N-S-	C-Fya-Jka-K-S-	4	N-	2	0.5
C-Fya-K-Kpa-	C-Fya-K-	142	Kpa-	2	0.01408
C-Fya-K-N-	C-Fya-K-	142	N-	2	0.01408
C-Cob-Fya-K-S-	C-Fya-K-S-	39	Cob-	1	0.02564
C-Fya-K-N-s-	C-Fya-K-s-	8	N-	4	0.5
C-Fya-N-S-	C-Fya-S-	2	N-	1	0.5
C-Jka-K-Lua-	C-Jka-K-	79	Lua-	5	0.06329
C-Jkb-K-Kpa-	C-Jkb-K-	74	Kpa-	3	0.04054
C-Jkb-K-N-S-	C-Jkb-K-S-	6	N-	1	0.16667
C-Cob-K-Kpa-	C-K-	294	Cob-Kpa-	1	0.0034
C-K-Lua-	C-K-	294	Lua-	1	0.0034
C-K-N-	C-K-	294	N-	1	0.0034
C-K-N-S-	C-K-S-	39	N-	4	0.10256
C-Kpa-S-	C-S-	4	Kpa-	1	0.25
E-Kpa-	E-	87	Kpa-	1	0.01149
E-Lua-	E-	87	Lua-	3	0.03448
E-Fya-Jka-K-Lua-	E-Fya-Jka-K-	69	Lua-	2	0.02899
E-Fya-Jkb-K-Lua-	E-Fya-Jkb-K-	96	Lua-	21	0.21875
E-Fya-K-Kpa-	E-Fya-K-	338	Kpa-	5	0.01479
E-Fya-K-Lua-	E-Fya-K-	338	Lua-	2	0.00592
Cob-E-Fya-K-S-	E-Fya-K-S-	55	Cob-	1	0.01818
E-Fyb-Jka-K-Kpa-	E-Fyb-Jka-K-	25	Kpa-	1	0.04
E-Fyb-Jka-K-Kpa-s-	E-Fyb-Jka-K-s-	3	Kpa-	2	0.66667
E-Fyb-K-Kpa-s-	E-Fyb-K-s-	7	Kpa-	1	0.14286
E-Fyb-K-N-S-	E-Fyb-K-S-	19	N-	1	0.05263
Cob-E-Jka-K-Kpa-	E-Jka-K-	270	Cob-Kpa-	1	0.0037
E-Jka-K-N-S-	E-Jka-K-S-	62	N-	4	0.06452

E-Jkb-K-Kpa-	E-Jkb-K-	159	Kpa-	20	0.12579
E-Jkb-K-Kpa-S-	E-Jkb-K-S-	55	Kpa-	10	0.18182
E-Jkb-K-Lua-S-	E-Jkb-K-S-	55	Lua-	1	0.01818
E-Jkb-K-N-S-	E-Jkb-K-S-	55	N-	1	0.01818
E-K-Kpa-	E-K-	1235	Kpa-	29	0.02348
E-K-Lua-	E-K-	1235	Lua-	9	0.00729
E-K-Lua-N-	E-K-	1235	Lua-N-	2	0.00162
E-K-N-	E-K-	1235	N-	6	0.00486
E-K-Kpa-S-	E-K-S-	80	Kpa-	18	0.225
E-N-S-	E-S-	12	N-	1	0.08333
Fya-Jka-K-Kpa-	Fya-Jka-K-	23	Kpa-	2	0.08696
Fya-Jka-K-N-	Fya-Jka-K-	23	N-	3	0.13043
Fya-Jka-K-Kpa-S-	Fya-Jka-K-S-	2	Kpa-	1	0.5
Fya-K-Kpa-	Fya-K-	334	Kpa-	11	0.03293
Fya-K-Lua-	Fya-K-	334	Lua-	2	0.00599
Fya-K-N-	Fya-K-	334	N-	2	0.00599
Jka-K-Kpa-	Jka-K-	301	Kpa-	1	0.00332
Jka-K-Lua-	Jka-K-	301	Lua-	1	0.00332
Jkb-K-Lua-	Jkb-K-	110	Lua-	10	0.09091
Jkb-K-Lua-S-	Jkb-K-S-	16	Lua-	1	0.0625
K-Kpa-	K-	204	Kpa-	100	0.4902
K-Lua-	K-	204	Lua-	41	0.20098
K-N-	K-	204	N-	5	0.02451
K-Kpa-M-S-	K-M-S-	14	Kpa-	1	0.07143
Kpa-[1]		0	Kpa-	2	0
Kpb-		0	Kpb-	1	0
Lua-		0	Lua-	3	0
N-		0	N-	7	0

[1] There is no extended phenotype information that can be used to reduce the search space for these last four antigen combinations.
