# Supplementary material for: Probabilistic mathematical modelling to predict the red cell phenotyped donor panel size
Source: PLoS One. 2022 Nov 11;17(11):e0276780. doi: 10.1371/journal.pone.0276780 (PMC9651592; doi:10.1371/journal.pone.0276780)
Supplement: Attachment — Submitted filename: Response to Reviewers.docx [file pone.0276780.s003.docx]

Dear Prof. Chalmers,

Thank you for the opportunity to resubmit the manuscript “*Probabilistic mathematical modelling to predict the red cell phenotype donor panel size*”. We would also like to thank the reviewer for his/her careful reading of the article and the insightful comments that we have addressed in full and have improved the presentation. Please see below for details where we have responded to the requirements of the journal and all the reviewer’s comments.

Journal Requirements:

1. Please ensure that your manuscript meets PLOS ONE's style requirements, including those for file naming. The PLOS ONE style templates can be found at

<https://journals.plos.org/plosone/s/file?id=wjVg/PLOSOne_formatting_sample_main_body.pdf> and <https://journals.plos.org/plosone/s/file?id=ba62/PLOSOne_formatting_sample_title_authors_affiliations.pdf>

*The aforementioned templates have been reviewed and the manuscript meets the requirements mentioned in them.*

2. In your Data Availability statement, you have not specified where the minimal data set underlying the results described in your manuscript can be found. PLOS defines a study's minimal data set as the underlying data used to reach the conclusions drawn in the manuscript and any additional data required to replicate the reported study findings in their entirety. All PLOS journals require that the minimal data set be made fully available. For more information about our data policy, please see <http://journals.plos.org/plosone/s/data-availability>.

"Upon re-submitting your revised manuscript, please upload your study’s minimal underlying data set as either Supporting Information files or to a stable, public repository and include the relevant URLs, DOIs, or accession numbers within your revised cover letter. For a list of acceptable repositories, please see <http://journals.plos.org/plosone/s/data-availability#loc-recommended-repositories>. Any potentially identifying patient information must be fully anonymized.

Important: If there are ethical or legal restrictions to sharing your data publicly, please explain these restrictions in detail. Please see our guidelines for more information on what we consider unacceptable restrictions to publicly sharing data: <http://journals.plos.org/plosone/s/data-availability#loc-unacceptable-data-access-restrictions>. Note that it is not acceptable for the authors to be the sole named individuals responsible for ensuring data access.

We will update your Data Availability statement to reflect the information you provide in your cover letter.

*The data set for this study is given in S1 Table in the appendix and there is no other data associated with this study. This data has been uploaded to the supplementary material file.*

3. We note you have included a table to which you do not refer in the text of your manuscript. Please ensure that you refer to Table 10 and 11 in your text; if accepted, production will need this reference to link the reader to the Table.

*Thank you for pointing this out. This material was additional analysis of the material given in the S1 Table and as such can be recovered from that table. Since we do not directly refer to these tables in the manuscript we have removed them.*


Response to Reviewer's Comments/Questions

5. Review Comments to the Author

Ln 121: I think, in fairness, that the paper by Blake and Clarke was primarily designed to evaluate the impact of frozen inventory.

*This has been noted in the text.*

Ln 129: Well, the multinomial distribution is just a generalization of the binomial distribution. I don't think independence vs. not independent is correct here. If we had 3 categories (X, Y, or Other) and compressed that to 2 (X and Other), we are still modelling the same selection process, we just have different categories for observation. Categories are mutually exclusive and if the number of categories is 2 we call it binomial and if 3 or more, it's multinomial. Same process, no difference in the idea of independence.

*Thanks. The text has been reworded here in line with comments.*

Ln 173: I understand the algorithm, of course, but a single donor is unlikely to be of great value, since the average transfusion request is typically greater than a single unit. So, the question then becomes one of having sufficient donors available (or units frozen) to meet an average demand with more availability being better.

*Additional text has been added here to clarify the intent of the text.*

Ln 238: Why m/2? Obviously this could be any fraction one likes, but why assume m/2? In particular, it appears later in the paper that 17% of donors don't return and 13% of new donors don't return. Could the authors comment and perhaps reconcile?

*Additional text has been added to clarify the choice of m/2.*

Figures 3 and 4: Could labels be added to axes of the graphs?

*Labels have been added as requested.*

Ln 325: Why 23541? The rationale for this number doesn't become apparent until much later.

*An explanation of this has been added to the text.*

Ln 334: There's a lot of variance, one would assume in the results. Why is an n of 3 sufficient to draw conclusions?

*The number of samples used was 10,000 however only three were displayed in the figures to highlight the variability that the Reviewer quite rightly points out.*

Figure 7: Data in Figure 7 is hard to decipher. Take antigen combination 3 in the chart. No target is shown (is it 0?). Consider also combination 4. Is there a target, but no simulation results? And also, when the authors say that the figure shows that a 95% CI is validated, how does one see this in the figure? Could a different method of displaying the data be considered?

*Figures 4, 5, 6 and 7 have been reordered and edited reducing the number of antigen combinations to improve readability, while still conveying the same information.*

Ln 370: It's confusing to talk about 10,000 simulations and the graph to show 3. Could this be changed to say 3 replications of 10,000 samples?

*The text has been reworded to clarify the stated information.*

Ln 375: Of course, this is one method for testing for sufficiency, but demand is likely to be less uniformly distributed over time than assumed.

*In validating the earlier results different scenarios for Monte Carlo sampling have been considered. Simulations have been run on the entire years data, simulations have been run on 15 non overlapping 8 day periods and 3 non overlapping 120 day periods, where 8 days and 120 days periods have been chosen based on LifeBlood procedures with respect to the storage of blood products and the returning donors. Since the 120 day simulation results were in line with all other results 3 consecutive periods was deemed to be enough. Where necessary donors can be approached to give blood 4 times a year.*

Table 5: Perhaps show values of K = 4 through 6 or 7 so that readers can get a sense of the improvement on the way to 95% rather than once exceeded.? Similar comments for 120 days.

*The tables have been recast to meet the Reviewers request.*
