# Supplementary material for: Probabilistic mathematical modelling to predict the red cell phenotyped donor panel size
Source: PLoS One. 2022 Nov 11;17(11):e0276780. doi: 10.1371/journal.pone.0276780 (PMC9651592; doi:10.1371/journal.pone.0276780)
Supplement: Attachment — Submitted filename: Response to Reviewers.docx [file pone.0276780.s004.docx]

RESPONSE TO REVIEWER’S REPORT

Again, we would like to thank the Reviewer for their careful reading of the manuscript and their insightful comments which have allowed us to improve our presentation.

We have addressed all comments made Reviewer and made changes as outlined below.

Pg 11: compatible RBC --> compatible RBCs

*Changed as required.*

Pg 15: blood donor phenotyped --> blood donors phenotyped

*Changed as required.*

Pg 18: Sentence starting "Of course, the one donor..." Honestly, I don't understand this sentence. Could it be reworded or expanded?

*The wording here has been changed as well taking into account the reviewers comments. The given sentence refers to the calculations within the simulation which is a modelled using a Mutlinomial distribution sampling*

Pg 18: I apologize - I probably should have asked this question in the first review. I don't understand what the authors mean when they say they say "the allocation of to a category is achieved by a random process based on a Multinomial distribution where S donors are allocated across r categories based on the category probabilities in Fig 2". Can the authors give more detail on how donors are allocated to categories? I'm struggling to think about how this would be implemented as Multinomial draw (which would require an enumeration of all possible values of n1, n2, ..., nr adding up to S). Is it a draw from a cumulative distribution for a donor that adds them to a category? Please more detail here, since it would be hard for others to reproduce the method without understanding this crucial step.

*Additional text has been added to clarify this situation.*

*In general terms this type of allocation problem arises frequently enough that a number of programming languages, including Python, Matlab and R provide implementations often based on acceptance/rejection sampling. For instance in Matlab it is implemented in the command mnrnd(S,p) where p is the vector of probabilities of categories with length r, the number of categories, and S is the number of donations.*

Pg 20, 1st Equation: Can you perhaps add more space between E[ni] and Var[ni]? The two look like a single equation.

*The equations have now been split across 2 lines.*

Pg 20, 2nd Equation: add a space between for and j

Pg 21, Equation under Theorem 2: What does the 2nd equation mean? Is it missing Ps? The first line appears (I didn't check the reference to Mallow) to set U and L bounds on Ps. So, what is the 2nd equation? Please clarify.

*We thank the referee for drawing our attention to this. We have taken the statement out of the equation and clarified the value of S_95_*

Pg 21 "within the given time periods". No mention of time periods has appeared in the paper to this point. You need to give the readers a "heads up" about this.

*The reference to time periods is not necessary here and will be dealt with later when they arise. So the statement has been removed*.

Pg 23: Sometimes called timelines, sometimes called timeframes. Please be consistent.

*We have changed all references to ‘timelines’*

Pg 23: Also, it's not clear to readers why timelines are introduced. Please give a sentence or two.

*The development of a timeline refers to the development of a testing schedule to meet request targets. Additional information has been given to make this clearer.*

Pg 24: The authors added a sentence about the selection of m/2 in the 1st equation on the page, but it doesn't answer my question. If m is the retention rate for donors, why is the retention rate m/2 for new donors? Do you mean that they are, on average, only in the system for 1/2 year? As it's written this says there is a different retention rate for new donors and that it's less than repeat donors. This is somewhat contradictory to most literature that says new donors have very poor retention rates. Regardless of the value used (m, m/2 or something different), the authors need to tell the readers why the value is an appropriate model for new donor retention.

*We thank the reviewer for drawing our attention to this. The calculations are consistent, but the wording has been improved to better reflect the underlying mathematics. Essentially, m=0.13 and, for example, there are around 35000 new donors phenotyped, a number that is currently increasing, and around 65600 already phenotyped. Using m/2 with M=q(1-m/2) gives the average number of donors donating during the year and thus the average supply*

Pg 33: Tables 5 and 6 have exactly the same titles. Please distinguish what's appearing in the table.

*The captions for Tables 5 and 6 have been updated to accurately distinguish between them.*

7. PLOS authors have the option to publish the peer review history of their article ([what does this mean?](https://journals.plos.org/plosone/s/editorial-and-peer-review-process#loc-peer-review-history)). If published, this will include your full peer review and any attached files.


*This will not be necessary.*
