# Supplementary material for: Probabilistic mathematical modelling to predict the red cell phenotyped donor panel size
Source: PLoS One. 2022 Nov 11;17(11):e0276780. doi: 10.1371/journal.pone.0276780 (PMC9651592; doi:10.1371/journal.pone.0276780)
Supplement: Attachment — Submitted filename: Response to Reviewers.docx [file pone.0276780.s005.docx]

Response to Reviewers

Again, we thank the referees for their comments and the opportunity to improve the paper. Here we have endeavoured to give fuller justifications for our choices and trust we have met the requirements to publish in Plos One.

We give responses to specific Reviewer comments in blue below.

**Reviewer #1:** Thank you for the revisions to the paper and the detailed work to address the items in R1.

I hate to do this, but I still don't understand the choice of m/2 as the retention rate for newly screened donors. The actual value (m/2) isn't important to the derivation that follows (it's part of the parameter M), but the selection of m/2 is not explained in the text.

To make matters worse, the explanation in the response to reviewers doesn't make sense to me and differs from what's written in the text.

The equation is:

D1 = (1-m)D0 + q(1-m/2)

Where:

Dy is the number of donors in the system in year y

q is the fraction of non-returning screened donors per annum as a decimal

Ok, so fine. The number of donors in D1 = Donors at D0 x retention rate + Screened donors x (a different retention rate)

It's not obvious why the non-screened donors have a greater likelihood of being retained than already screened donors.

The text says that m/2 is chosen as the mean of a uniform distribution of donors throughout the year. However, the note to reviewers says m = 0.13 and says "using m/2 gives the average number of donors donating during the year and thus the average supply".

Neither of these two explanations really is clear. I think what's meant here is that there are q donors screened over the course of the year and thus, on average, a newly screened donor has been registered for six months at the end of the first year. Accordingly, the non-return rate has been pro-rated by m/2 to reflect this fact.

Could the wording perhaps be changed to make this clear? Otherwise the selection of m/2 looks arbitrary. Given that it disappears in the following line into M, the reader is left wondering why the parameter was changed.

Finally, if an empirical value of m can be found (m = 0.13), then it would seem to me that an empirical value of m' for new donors could be found from data and the assumption of m/2 avoided (or perhaps confirmed).

We thank Reviewer 1 for his comments on the calculations involving m/2 and have inserted the suggested wording into the text.

**Reviewer #2:** The authors present results from a probabilistic model to meet requests for specific antigen combinations in Australia. The authors use data from Lifeblood (the sole provider for fresh blood components to Australians) to understand distributions of requests across antigen combinations and specific targets over time periods. The authors are able to estimate total donor sizes needed to fulfill requests with probability 0.95 and to estimate the length of time (and percentage of geno- or pheno-typing) it would take to achieve the necessary number of geno- (or pheno-) typed samples. The manuscript will be strengthened if the authors address the following points.

1. In the Abstract (line 12-14), authors state that simulations were used to attain a 95% confidence interval, but that doesn't seem to be the case. Simulations appear to have been run to estimate a minimum success percentage. No confidence intervals are presented.

We thank the Reviewer for this comment and have appropriately changed all instances to ``success percentage’’.

2. On page 10, lines 1-8 (describing Figure 2), I am a bit confused. Figure 2 appears to show the distribution of the requests for certain genotype antigen combinations based on historical Lifeblood data. These probabilities then seem to be used as probabilities that donors will have a particular genotype-antigen combination. This may be the best the authors can do, but I think it warrants a statement possibly in the methods, but also likely as a limitation in the Conclusions section, that request probabilities may not be representative of the distribution of donors. If I am misunderstanding something, authors should clarify.

This point has been addressed when we address point 18 and have included information on the limitations and strengths of the model.

3. On pages 12 and13, the notation is a bit confusing, since authors start using n_j as a random variable, by giving its expected value and variance and using it in the probability expression on line 4 of page 13. However, on page 11, authors use n_j to refer to the "observed" number of requests (used in the multinomial probability expression). Although minor, authors might consider distinguishing between the random variable and the observed value (often, in probability, capital letters refer to a random variable and lower-case letters refer to the observed value).

We acknowledge the Reviewers comments, however we feel that given the context the meaning is clear enough and prefer not to change it at this late stage.

4. Also on page 13, line 5, authors state an assumption that n_j >= k_j. The probability in the previous line is for the probability that all n_i>=k_i, so if that is assumed, then the probability calculation isn't all that interesting. I don't believe authors need to state n_j>= k_j in line 5.

As suggested by the Reviewer this information has been removed.

5. In Theorem 2, do authors really need the 2nd bound given in line 17? As written, it is not all that interesting (I actually think there is something incorrect here since Mallow's paper has something different, even when translated to the direction of inequalities used here). As written, the smallest line 17 can be is 1 and the largest it can be is e^r. We already know that probabilities have to be less than or equal to 1, so it really doesn't add any information.

When the lower bound is greater than or equal to 0.95, then the difference between the upper and lower bound is extremely small, so that the lower bound is a good estimate of the probability and can be calculated directly.

6. The reference for the proof to Theorem 2, I think should be reference #11 not #10

Corrected as given.

7. In response to another reviewer, authors added a definition of S_95 on line 10, page 14, but I actually found this a bit confusing, since it suggests authors estimate the n_j. Assuming, I am understanding the steps authors took to estimate S_95, authors only need the p_j (which are from Figure 2, supplemental table), k_i (which I'm assuming are also from the supplemental table) and then they can perform the probability calculations in the lower bound at different S to find the S that gets a lower bound greater than 0.95. If the steps are actually different, authors should clarify what is done (they may want to clarify even if what I describe is correct, since not everyone will be able to follow what is currently written).

We thank the Reviewer for this comment and have removed the summation.

8. On page 15, lines 1-5 are a bit confusing, especially the calculation of the minimum percentage of success in attaining the targets. How can the number of failures to meet targets be calculated as a percentage of the number of categories r (unless this is done for each of the 10000 samples)? Is a failure at the sample level (so any sample that does not meet targets) or at the category level across the samples, so the total number of categories across the 10000 samples for which the targets were not met? Some clarification is needed.

Addtional wording has been given to clarify this point.

9. Authors use "confidence level" in a lot of different places (including line 7, page 15, Tables 3 and 4 and related text), but this term is used specifically in statistics for something other than what it seems the authors are doing (unless authors are calculating minimum percentage of success differently than stated). Authors, in some cases (such as Tables 3 and 4) are really just talking about a probability (or lower bound of a probability).

This has been addressed as in point 1 above.

10. The added explanation for m/2 in line 5 page 16 is still not clear. Why does "m" impact the number of new donors? Authors say that m/2 is from the Uniform, which would suggest they are working with a Uniform distribution on (0, m), but it isn't clear why that would make sense for number of donors. Authors need to provide more explanation.

This has been addressed as stated in the response to Reviewer 1.

11. lines 2 and 11 on page 20 (and titles to Tables 3 and 4): this may be related to my point #7 above, but authors refer to "to obtain at least one donor for each...". That would suggest that k_i in the equation in line 9, page 14, is equal to 1 for all i. Is that really what is being done?

Yes, this is correct as stated in a number of places in the article.

12. When authors refer to 3 sampling scenarios in Figures 4-7, those are just 3 different multinomial samples from a particular multinomial distribution right? They aren't different scenarios...

Three specific different instances of request data were used to set data and to validate the theoretical results. So, three different samples have been used.

13. page 22, lines 2-4: what is meant by the number being estimated by Mallows' bounds (again, this could be due to my confusion in point 7)? Is something different done here to get 23541 (which I assume is what was estimated) maybe based on different targets than what were used in calculations for Table 3?

Additional wording has been added in the text to clarify this.

14. page 22, line 4: "The target data was" should be "The target data were"

Corrected as stated.

15. Authors should clarify the time periods used for Tables 5 and 6. In particular, I'm a bit confused how the consecutive time periods come in - is there something different in saying targets are based on 15 consecutive 8-day periods versus targets were based on 144 consecutive days of requests, for example, or was there something distinct about the 8-day (or 120-day) periods that were somehow used?

As stated in the text 8 days was chosen as it is the preferred turn over period for fresh blood.

16. Table 5: Why is the first entry for S=0 under "Targets based on 8-day periods"?

This indeed was a typo and has been corrected.

17. It is not clear why authors feel that the multinomial simulations validate the numbers calculated using the Mallows' bound, especially for phenotyping, where Table 4 suggests 188,000 donors need to be phenotyped, while Table 6 has 95% probability at about half that size (94,156). Yes, the Mallows' bound value will exceed 95% probability based on the simulations, but that is potentially quite a bit more phenotyping than would be needed based on the simulations.

The text below has been added to further clarify the use of the given model. In addition, the analysis around tables 4, 5 and 6 was added as a cross validation for results based on the available data. The choice of the time periods is to match Lifeblood’s actual procedures, and this had already been noted in the text. The difference in the estimates is due to a confounding of the rare event probabilities the shorter time periods and the 95% success rate. The text that has been added in the discussion of the model on page 15 is copied below.

It is well known that one area in which standard Monte Carlo simulations do not work particularly well are in rare event simulation [10]. In this case many simulations may be needed to estimate these rare events, and this can become computationally extremely burdensome. New approaches have been developed such as the cross-entropy method [10] that can be represented as a stochastic search algorithm that can address the issue of rare event estimation, but implementations are not trivial. However, in our case we have access to tight upper and lower bounds on the multinomial probability of meeting targets with high probability through the theory of Mallows. Thus rare event estimation of rare antigen combinations is just a matter of computing marginal (binomial) probabilities and avoids the issues associated with rare event simulation through Monte Carlo approaches.

[10] R.V. Rubinstein, D.P. Kroese, Simulation and the Monte Carlo Method, John Wiley and Sons, 2008.

18. Authors should provide some discussion of strengths and limitations in their conclusions.

A discussion of strengths and limitations have been added to the conclusion.

7. PLOS authors have the option to publish the peer review history of their article ([what does this mean?](https://journals.plos.org/plosone/s/editorial-and-peer-review-process#loc-peer-review-history)). If published, this will include your full peer review and any attached files.


If you choose “no”, your identity will remain anonymous, but your review may still be made public.


**Do you want your identity to be public for this peer review?** For information about this choice, including consent withdrawal, please see our [Privacy Policy](https://www.plos.org/privacy-policy).

Reviewer #1: No

Reviewer #2: No
