# Supplementary material for: Probabilistic mathematical modelling to predict the red cell phenotyped donor panel size
Source: PLoS One. 2022 Nov 11;17(11):e0276780. doi: 10.1371/journal.pone.0276780 (PMC9651592; doi:10.1371/journal.pone.0276780)
Supplement: Attachment — Submitted filename: Response to Reviewers.docx [file pone.0276780.s006.docx]

Response to Reviewers

Thank you for the opportunity to resubmit the manuscript “Probabilistic mathematical modelling to predict the red cell phenotype donor panel size”. We would also like to thank the Reviewers for their careful reading of the article and their insightful comments.

We unreservedly apologize to Referee #2 for our failure to make their important correction to our statement of Theorem 2.

Please see below for details where we have responded to the requirements of the journal and the reviewers’ comments; we list only the outstanding issues. We give responses to specific Reviewer comments in blue below.

Response to the Reviewers:

5. Is the manuscript presented in an intelligible fashion and written in standard English?

PLOS ONE does not copyedit accepted manuscripts, so the language in submitted articles must be clear, correct, and unambiguous. Any typographical or grammatical errors should be corrected at revision, so please note any specific errors here.

Reviewer #1: No

Reviewer #2: Yes

RESPONSE:

We believe, like Reviewer #2, that the manuscript “is presented in an intelligible fashion and written in standard English”. Moreover, Reviewer #1 has previously answered “Yes” to this question and has not presented specific criticisms for us to address.

6. Review Comments to the Author

Please use the space provided to explain your answers to the questions above. You may also include additional comments for the author, including concerns about dual publication, research ethics, or publication ethics. (Please upload your review as an attachment if it exceeds 20,000 characters)

Reviewer #1: (No Response)

Reviewer #2: The authors have addressed the majority of my earlier concerns. The one remaining point has to do with the statement of Theorem 2, giving the bounds. I had raised this issue before and the author's response does not address my comment. The 2nd upper bound (given in line 17 on page 13) does not appear to be correct (looking at Mallows' paper, the exponent has a negative sign and the direction of the inequality also should be switched). The bound, as written, is not interesting. The exponent (sum of the probabilities), as written, has a minimum of 0, which means the smallest this upper bound can be is 1 (e^0=1). Since probabilities have to be less than or equal to 1, this bound, as written, does not really give us any information. I'm not even sure if this is the upper bound authors use in tables 3 and 4 or if they use the upper bound from line 16 (product of the probabilities). Authors need to either correct line 17 or remove it.

RESPONSE:

We unreservedly apologize to Reviewer #2 for failing to correct this misprint

The inequalities in Theorem 2 now read

$$1-\sum_{j=1}^{r} Prob({n_{j}<k_{j})\leq P}_{S}\leq\prod_{j=1}^{r} Prob\left( n_{j}\geq k_{j} \right)\leq exp\left( -\sum Prob\left( n_{j}<k_{j} \right) \right).$$

Further Mallows states the inequality

$1-\sum_{j=1}^{r} Prob({n_{j}<k_{j})\leq P}_{S}\leq\prod_{j=1}^{r} Prob\left( n_{j}\geq k_{j} \right)$

which we use in our paper and references Esary JD, Proschan F, Walkup DW. Thus, we have included this paper in our References and changed the first citations of the Mallows paper to “Mallows (see also Esary, Proschan and Walkup)”.

In particular, we used $\prod_{j=1}^{r} Prob\left( n_{j}\geq k_{j} \right)$ and the **R** language to compute the upper bounds presented in the paper. We checked our calculations by separately computing $exp\left( -\sum Prob\left( n_{j}<k_{j} \right) \right)$ using **MATLAB** although we didn’t report these values.
